# Nonlinear structure-extended cavity interaction simulation using a new version of harmonic balance method

**DOI:** 10.1371/journal.pone.0199159

**Published:** 2018-07-03

**Authors:** Yiu-Yin Lee

**Affiliations:** Department of Architecture and Civil Engineering, City University of Hong Kong, Kowloon Tong, Kowloon, Hong Kong; Hunan University, CHINA

## Abstract

This study addresses the nonlinear structure-extended cavity interaction simulation using a new version of the multilevel residue harmonic balance method. This method has only been adopted once to solve a nonlinear beam problem. This is the first study to use this method to solve a nonlinear structural acoustic problem. This study has two focuses: 1) the new version of the multilevel residue harmonic balance method can generate the higher-level nonlinear solutions ignored in the previous version and 2) the effect of the extended cavity, which has not been considered in previous studies, is examined. The cavity length of a panel-cavity system is sometimes longer than the panel length. However, many studies have adopted a model in which the cavity length is equal to the panel length. The effects of excitation magnitude, cavity depth, damping and number of structural modes on sound and vibration responses are investigated for various panel cases. In the simulations, the present harmonic balance solutions agree reasonably well with those obtained from the classical harmonic balance method. There are two important findings. First, the nonlinearity of a structural acoustic system highly depends on the cavity size. If the cavity size is smaller, the nonlinearity is higher. A large cavity volume implies a low stiffness or small acoustic pressure transmitted from the source panel to the nonlinear panel. In other words, the additional volume in an extended cavity affects the nonlinearity, sound and vibration responses of a structural acoustic system. Second, if an acoustic resonance couples with a structural resonance, nonlinearity is amplified and thus the insertion loss is adversely affected.

## 1 Introduction

In recent decades, many researchers have tackled various vibro-acoustic and fluid-structure interaction problems (e.g., [1–8]). Most of these studies that have included sound and vibration analyses of a panel backed by a cavity have concerned the transmission losses of various enclosure panels and have adopted the assumption that the cavity size is equal to the panel size. For example, Pan et al. [9], and Nehete et al. [10] considered a structure coupled with a cavity and subjected to external excitation. The logarithm difference between the external and internal sound pressures is defined as the transmission loss. In fact, the insertion loss of a panel is a very practical indicator and is defined as the logarithm difference between the sound pressures with and without the panel. This can truly reflect the sound reduction ability. In practice, the cavity length of a panel-cavity system is sometimes longer than the panel length, which may significantly affect sound reduction, but has not been considered in the aforementioned work. Although very few studies have examined the nonlinear structural acoustic problem considered in this study, numerous researchers have studied linear structural acoustic or nonlinear structure problems (e.g., [11–18]). The only research work directly related to this study of a nonlinear panel coupled with an extended cavity was done by Lee [19], who investigated the free vibration of a panel backed by an extended cavity only. It did not contain any new solution method and considered the forced vibration and sound responses. Nonlinear vibration (or large amplitude vibration) must be considered in structural acoustic problems for two reasons. First, when a thin panel is used, it vibrates nonlinearly because its weak structural stiffness can cause large amplitude vibrations. Second, a panel mounted very close to a sound source may also vibrate nonlinearly due to the high excitation level imposed by the sound source.

**Fig 1:**
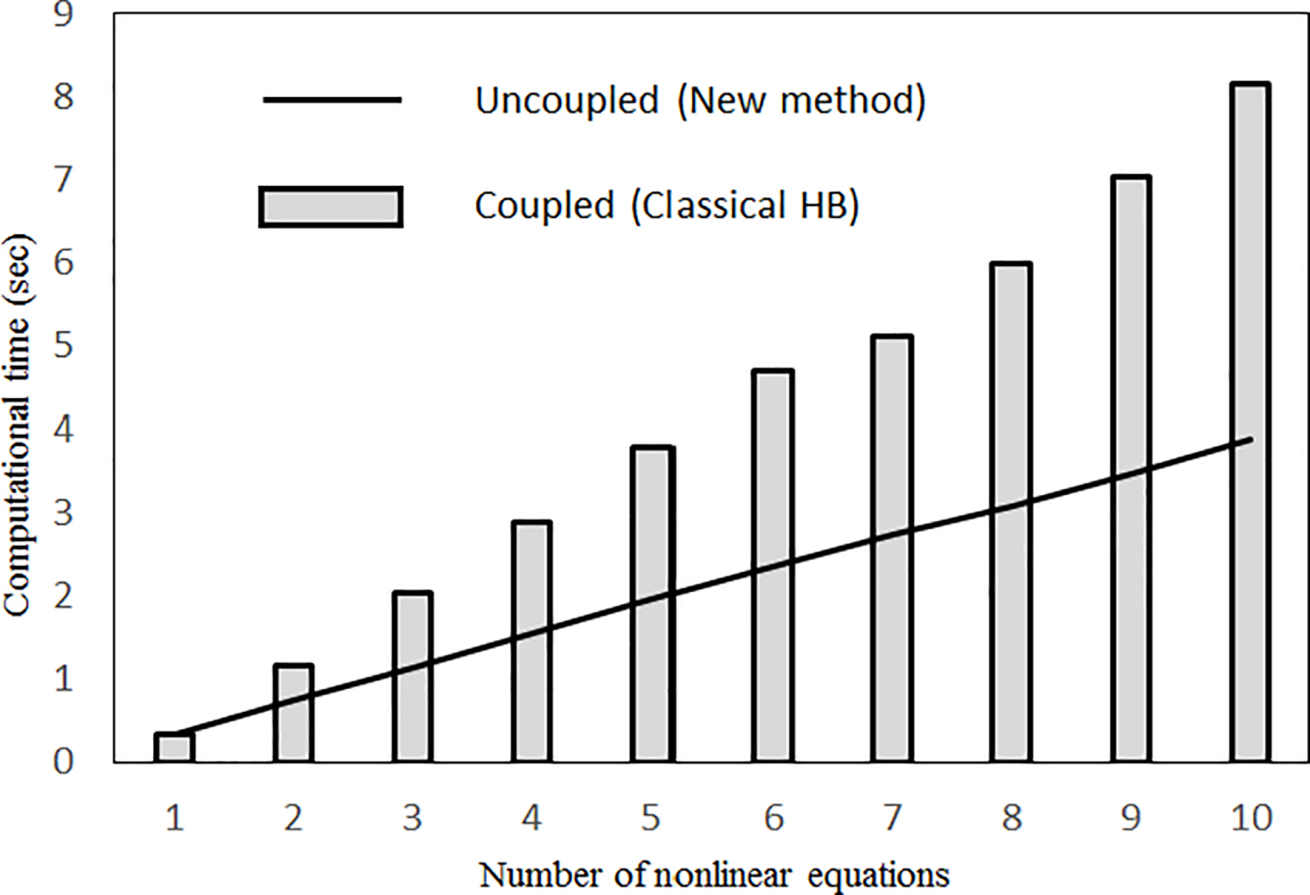
Computational time for solving nonlinear algebraic equations generated in the problem (single mode, no damping).

Many studies have applied various solution methods (e.g., the perturbation method, multiple scales method and elliptic integral method) to various nonlinear vibration/oscillation problems or differential equations (e.g., [20–26]). The total classical harmonic balance method and the incremental harmonic balance method have also been commonly used to solve nonlinear problems (e.g., [27–31]). Because the incremental harmonic balance method eliminates all nonlinear terms during the variational process, some nonlinear behaviour types are missing. The classical harmonic balance method retains all of the nonlinear terms to produce the multiple solutions possible in a set of nonlinear algebraic equations. The nonlinear solutions are assumed by Fourier series expansion to produce a set of nonlinear algebraic equations. From the results of Srirangarajan [27], the results from the classical harmonic balance method closely agreed with those of the closed-form solution. However, it is extremely time consuming to use the classical harmonic balance method to construct higher-order analytical approximations. Hence, in this study, the other harmonic balance method (i.e., the multilevel residue harmonic balance method), which was developed by Leung and Guo [32] and by Hansan et al. [33], is modified and used to solve the nonlinear structural acoustic problem. The main advantage of this method is that the higher-level solutions to any desired accuracy can be obtained easily by solving only one nonlinear algebraic equation and a set of linear algebraic equations in each solution level. Table 1 shows the numbers of algebraic equations required by the three harmonic balance methods to solve the governing equation of a single-mode cubic nonlinear undamped panel vibration. In the previous multilevel residue harmonic balance method, the higher-level nonlinear phenomena are omitted because no nonlinear algebraic equations are required to calculate the higher-level harmonic components. The new multilevel residue harmonic balance method requires only one nonlinear algebraic equation at each solution level [34]. Fig 1 shows that the computational effort required for the new method is less than that for the classical harmonic balance method, which generates more coupled nonlinear algebraic equations. Tables 2 and 3 shows the comparison between the numbers of nonlinear terms generated in the two harmonic balance methods. There are much more nonlinear terms in the classical harmonic balance method. In fact, in the total time of the entire solution process should include the time spent on the formulation derivation as well as the time on the computation. The more nonlinear terms are generated, the more time is spent on the formulation derivation. Unlike finding the roots of the nonlinear algebraic equations (which is done by computer), the formulation derivation and setup of initial guess value for nonlinear algebraic equations are done manually. In this study, only two or three nonlinear algebraic equations are considered for each case. Nowadays, personal computers are very fast so that the computational time is not the bottleneck in the entire solution process and much less than the times spent on the manual steps, which depend on the complexity of the algebraic equations.

**Table 1:** Numbers of algebraic equations for a single mode cubic nonlinear undamped panel vibration.

	Zero level solution		1^st^ level solution		2^nd^ level solution	
	Nos. of uncoupled nonlinear algebraic equations	Nos. of linear algebraic equations	Nos. of uncoupled nonlinear algebraic equations	Nos. of linear algebraic equations	Nos. of uncoupled nonlinear algebraic equations	Nos. of linear algebraic equations
*Old multi-level residue HB method*	1	0	1	2	1	3
*New multi-level residue HB method*	1	0	2	1	3	2
	One harmonic term Nos. of coupled nonlinear algebraic equations		Two harmonic terms Nos. of coupled nonlinear algebraic equations		Three harmonic terms Nos. of coupled nonlinear algebraic equations	
*Classical* *HB method*		1		2		3

**Table 2:** Number of nonlinear terms in the algebraic equations generated in the problem (no damping, new multi-level residue HB method).

	Zero level solution	1^st^ level solution	2^nd^ level solution
One mode	1	3	5
Two modes	2	6	10
Three modes	3	9	15

**Table 3:** Number of nonlinear terms in the algebraic equations generated in the problem (no damping, classical HB method).

	One harmonic term	Two harmonic term	Three harmonic term
One mode	1	6	18
Two modes	2	12	36
Three modes	3	18	54

Using the classical approach and modal analysis, the nonlinear modal governing equations are developed to represent the large-amplitude structural vibration of an enclosure panel coupled with a cavity. Then, the proposed harmonic balance method is used to solve the nonlinear differential equations. Generally, the classical approach is used for those structures and acoustic cavities with regular geometries (e.g. rectangle and circle). In fact, there were some finite element methods developed recently for structural/acoustic problems (e.g. [35–37]). They are more suitable for handling models with complicated geometries and boundary conditions. If one of these finite elements is used for the problem in this study, a set of nonlinear differential equations will also be derived from the finite element process. The proposed harmonic balance method is still suitable for solving them.

## 2 Theory

**Fig 2:**
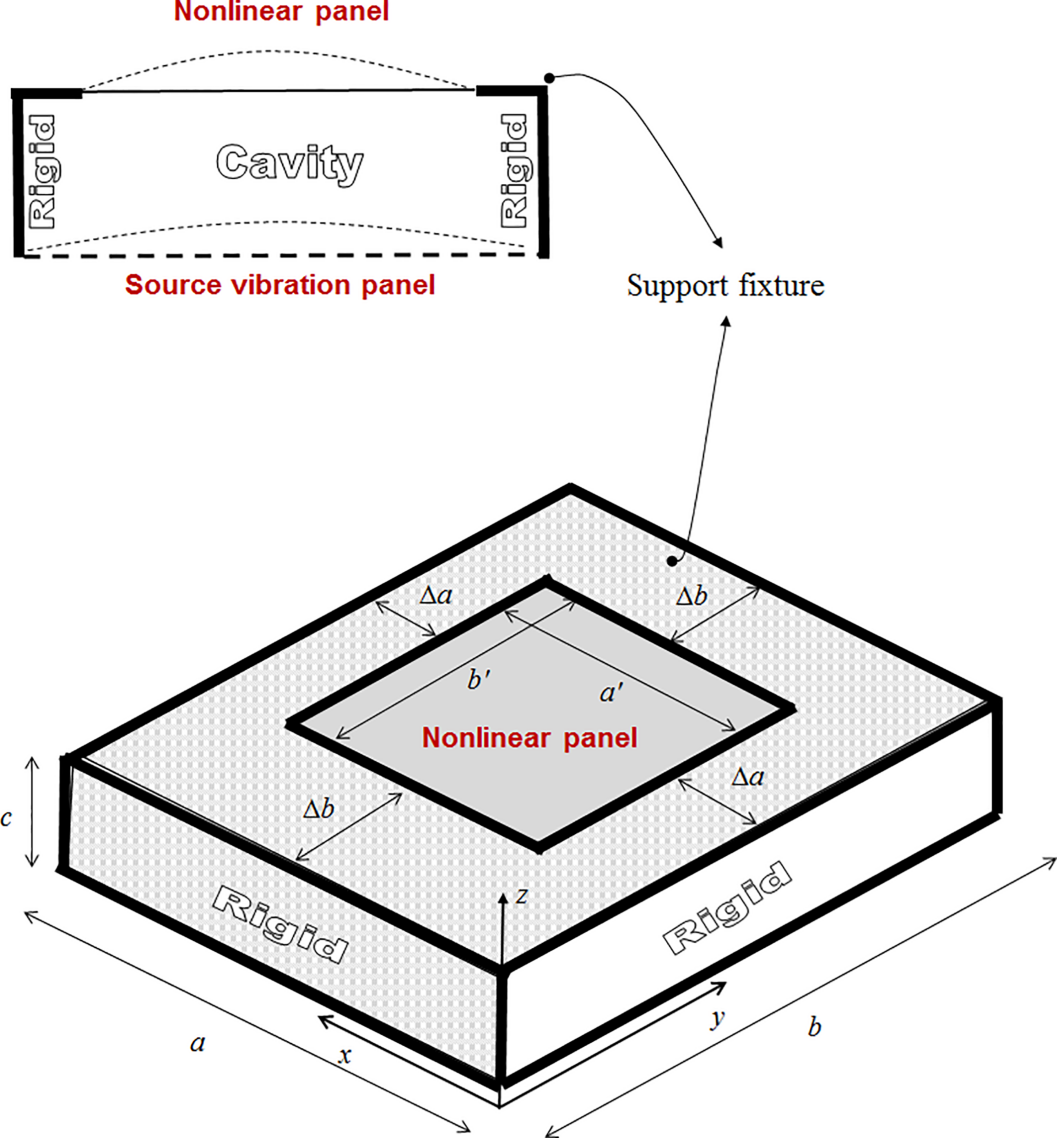
A nonlinear panel coupled with extended cavity.

Fig 2 shows a nonlinear panel backed by an extended cavity. The cavity walls are acoustically and structurally rigid. The excitation source is the vibrating panel, which induces the acoustic pressure force within the cavity. This section mainly describes the deduction of the formula to calculate the acoustic pressures within the cavity and the panel vibration responses, which are used to find the insertion loss of the nonlinear panel. Eq (1) is the governing equation [2] of the acoustic pressure within a rectangular cavity, as shown in Fig 2.

$$(\nabla^{2}+k_{h}^{2})p_{Q,h}(x,y,z)=0$$ (1)

where *k*_*h*_ is the wave number; *p*_*Q*,*h*_*(x*,*y*,*z)* is the acoustic pressure at the position of *(x*,*y*,*z)*; and *Q* and *h* represent the panel mode number and harmonic component order, respectively.

The total acoustic pressure field is assumed to be equal to the sum of the modal pressure fields:

$$p_{Q,h}(x,y,z)=\sum_{J=1}^{\bar{J}}P_{QJ,h}\varphi_{J}(x,y,z)$$ (2)

where *P*_*QJ*,*h*_ is the acoustic pressure amplitude of the *J*^th^ acoustic mode and $\bar{J}$ is the number of acoustic modes used. *φ*_*J*_ is the *J*^th^ acoustic mode function, which is expressed as follows:

$$\varphi_{J}(x,y,z)=cos\frac{l_{x}\pi x}{a}cos\frac{l_{y}\pi y}{b}cos\frac{l_{z}\pi z}{c}$$ (3)

where *l*_*x*_, *l*_*y*_ and *l*_*z*_ are even non-negative integers and *a*, *b* and *c* are the cavity dimensions in the *x*, *y* and *z* directions, respectively.

Using the technique of integration by parts in [38], Eq (1) can be expressed in the following form:

$$(k_{h}^{2}-k_{J}^{2})\alpha_{JJ}P_{QJ,h}=-\int_{z=0}^{}\frac{\partial p_{Q,h}}{\partial n}\varphi_{J}dxdy+\int_{z=c}^{}\frac{\partial p_{Q,h}}{\partial n}\varphi_{J}dxdy$$ (4)

where

$k_{J}^{}=\sqrt{{\left(\frac{l_{x}}{a}\right)}^{2}+{\left(\frac{l_{y}}{b}\right)}^{2}+{\left(\frac{l_{z}}{c}\right)}^{2}}$
$\alpha_{JJ}=\int_{vol}^{}\varphi_{J}\varphi_{J}dxdydy$

The derivative term on the right side of Eq (4) is the pressure gradient on the source panel or the nonlinear panel, which are expressed as follows:

At *z* = 0,

$$\frac{\partial p_{Q,h}}{\partial n}=-i\rho {}_{o}h\omega v_{o}(x,y)$$ (5)

$$v_{o}\left(x,y\right)=ih\omega w_{o}\left(x,y\right)$$ (6)

At *z* = *c*, *for Δa +a’ > x > Δa* and *Δb +b’ > y > Δb*,

$$\frac{\partial p_{Q,h}}{\partial n}=-i\rho {}_{o}h\omega v_{Q}(x,y)$$ (7)

$$v_{Q}(x,y)=ih\omega w_{Q}(x,y)$$ (8)

Otherwise,

$$\frac{\partial p_{Q,h}}{\partial n}=v_{Q}(x,y)=0$$ (9)

where *ρ*_o_ is the air density; *Δa* = *a—a’* and *Δb* = *b—b’*; and *v*_*o*_(*x*,*y*) and *v*_*Q*_(*x*,*y*) are the normal velocities of the source panel and the nonlinear panel, respectively. *w*_*o*_(*x*,*y*) and *w*_*Q*_(*x*,*y*) are the displacements of the source panel and the nonlinear panel, respectively, which are expressed as follows:

$$w_{o}(x,y)=A_{o}\phi_{o}(x,y)$$ (10)

$$w_{Q,h}(x,y)=A_{Q,h}\phi_{Q}(x,y)$$ (11)

where *A*_*o*_ is the vibration amplitude of the source panel;*ϕ*_*o*_*(x*,*y)* is the mode shape of the source panel and assumed as a double sine function (i.e. sin (*πx/a’*) sin (*πy/b’*); *ϕ*_*Q*_*(x*,*y)* is the *Q*^th^ mode shape of the panel at *z* = *c* and also assumed as a double sine function (i.e. sin (*mπ(x- Δa)/a’*)sin (*nπ(x- Δb)/b’*)); and *m* and *n* are the panel mode numbers. *A*_*Q*,*h*_ is the *h*^th^ harmonic component of the panel vibration amplitude and equal to the sum of the *h*^th^ harmonic components of the vibration amplitudes at different solution levels:

$$A_{Q,h}={A0}_{Q,h}+{A1}_{Q,h}+{A2}_{Q,h}+\cdots$$ (12)

By substituting Eqs (5–11) into Eq (4), the acoustic pressure amplitude can be expressed as follows:

$$P_{QJ,h}=\frac{-{\rho_{o}(h\omega )}^{2}}{(k_{h}^{2}-k_{J}^{2})\alpha_{JJ}}(\alpha_{QJ}A_{Q,h}-\alpha_{oJ}A_{o})$$ (13)

where

$\alpha_{QJ}=\int_{z=c}^{}\phi_{Q}\varphi_{J}dxdy$
$\alpha_{oJ}=\int_{z=o}^{}\phi_{o}\varphi_{J}dxdy$.

Then, the modal contribution of the acoustic pressure acting on the panel is expressed as follows:

$$F_{Q}^{}=\sum_{h=1,3,5\ldots }^{\bar{H}}\sum_{J=1}^{\bar{J}}\frac{\alpha_{QJ}}{\alpha_{QQ}}P_{QJ,h}$$ (14)

where

$\alpha_{QQ}=\int_{z=c}^{}\phi_{Q}\phi_{Q}dxdy$
  $\bar{J}=number\;of\;acoustic\;modes\;used$

$\bar{H}=number\;of\;harmonic\;terms\;used$

Hence, the modal governing equation of the nonlinear panel, which is directly excited by the interior acoustic pressure force and expressed as follows [2,19]:

$$\rho \frac{d^{2}A_{Q}}{dt^{2}}+\rho \omega_{Q}^{2}A_{Q}+\beta_{Q}{A_{Q}}^{3}=F_{Q}^{}sin(\omega t)$$ (15)

where *A*_*Q*_ and *ω*_*Q*_ are the modal amplitude and resonant frequency of the *Q*^th^ panel mode, respectively; *m* and *n* are the panel mode numbers; *a’* is the panel length; *γ* is the aspect ratio; *E* is Young’s modulus; *ν* is Poisson’s ratio; *τ* is the panel thickness; *ρ* is the panel surface density; *F*_*Q*_ is the modal force magnitude; and *ω* is the excitation frequency, and *β*_*Q*_ is the nonlinear stiffness coefficient which is given by

$$\beta_{Q}=\frac{E\tau }{4(1-v^{2})}{\left(\frac{m\pi }{a\prime }\right)}^{4}\left[(1+{\left(\frac{n}{m}\gamma \right)}^{4})(\frac{3}{4}-\frac{v^{2}}{4})+v{\left(\frac{n}{m}\gamma \right)}^{2}\right]$$ (16)

The proposed solution method [34] was newly presented for nonlinear beam problem in 2017, modified from the previous harmonic method in [32,33], is applied to the nonlinear forced vibration of a flexible panel. The solution form of *A*_*Q*_ is expressed as follows:

$${A_{Q}=\varepsilon^{0}A0}_{Q}+{\varepsilon^{1}A1}_{Q}+\varepsilon^{2}{A2}_{Q}+higher\;order\;terms$$ (17)

$${A0}_{Q}={A0}_{Q,1}sin(\omega t)$$ (18)

$${A1}_{Q}={A1}_{Q,1}sin(\omega t)+{A1}_{Q,3}sin(3\omega t)$$ (19)

$${A2}_{Q}={A2}_{Q,1}sin(\omega t)+{A2}_{Q,3}sin(3\omega t)+{A2}_{Q,5}sin(5\omega t)$$ (20)

$$A_{Q,1}={A0}_{Q,1}+{A1}_{Q,1}+{A2}_{Q,1}+higher\;order\;terms$$ (21)

$$A_{Q,3}={A1}_{Q,3}+{A2}_{Q,3}+higher\;order\;terms$$ (22)

$$A_{Q,5}={A2}_{Q,3}+higher\;order\;terms$$ (23)

where *ε* is an embedding parameter, the terms associated with *ε*^*0*^, *ε*^*1*^ and *ε*^*2*^ represent the zero, 1^st^ and 2^nd^ levels terms, respectively; *A0*_*Q*_, *A1*_*Q*_ and *A2*_*Q*_ are the zero, 1^st^ and 2^nd^ level panel responses, respectively; *A0*_*Q*,*1*_, *A1*_*Q*,*1*_ and *A2*_*Q*,*1*_ are the zero, 1^st^ and 2^nd^ level amplitudes of the sin(*ωt*) responses, respectively; *A1*_*Q*,*3*_ and *A2*_*Q*,*3*_ are the 1^st^ and 2^nd^ level amplitudes of sin(3*ωt*) responses, respectively; *A2*_*Q*,*5*_ is the 2^nd^ level amplitude of the sin(5 *ωt*) response; and *A*_*Q*,1_, *A*_*Q*,3_ and *A*_*Q*,5_ are the sin(*ωt*), sin(3 *ω t*) and sin(5 *ω t*) components of the modal amplitude.

Substitute Eq (17) into Eq (15) and collect the terms associated with *ɛ*^*0*^ to set up Eq (24). Then, consider the harmonic balance of sin(*ωt*) to set up Eq (26) and find the zero level solution to *A*0_*Q*,*1*_

$$\rho \frac{d^{2}{A0}_{Q}}{dt^{2}}+\rho \omega_{Q}^{2}{A0}_{Q}+\beta_{Q}{{A0}_{Q}}^{3}-F_{Q}^{}sin(\omega t){=R0}_{Q}(t)$$ (24)

$$\int_{0}^{2\pi }{R0}_{Q}(t)\sin\left(\omega t\right)dt=0$$ (25)

$$-\rho \omega^{2}{A0}_{Q,1}+\rho \omega_{Q}^{2}{A0}_{Q,1}+\frac{3}{4}\beta_{Q}{{A0}_{Q,1}}^{3}=F_{Q}^{}$$ (26)

where *R*0_*Q*_ is the sum of all terms associated with *ɛ*^*0*^

Rewrite Eq (26) as the following cubic algebraic equation form in terms of one unknown only

$${Z0}_{1}{A0}_{Q,1}+{Z0}_{3}{{A0}_{Q,1}}^{3}={Z0}_{0}$$ (27)

where *Z*0_*1*_ and *Z*0_*3*_ are the coefficients that precede the linear and nonlinear terms in Eq (26) and *Z*0_*0*_ is the constant term.

If damped cases are considered, rewrite Eq (27) to obtain the following equation:

$${A0}_{Q,1}=\frac{{Z0}_{0}}{{Z0}_{1}+{Z0}_{3}{{A0}_{Q,1}}^{2}}$$ (28)

$$(C0+iD0)=\frac{{Z0}_{0}}{{Z0}_{1}+{Z0}_{3}({C0}^{2}-{D0}^{2})+i(2{\xi \omega \omega }_{po}+2{Z0}_{3}C0D0)}$$ (29)

where *C0* and *D0* are the real and imaginary parts of *A*0_*Q*,1_, respectively, and *ω*_*po*_ is the resonant frequency of the zero-level resonance.

The zero-level resonance occurs when the magnitude of *A*1_*Q*,3_ is maximum and *ω* = *ω*_*po*_. Thus, the expression *Z*0_1_ + *Z*0_3_(*C*0^2^−*D*0^2^), which is function of *ω*, is equal to 0.

$${Z0}_{1}+{Z0}_{3}({C0}^{2}-{D0}^{2})=0$$ (30)

As *Z*0_1_ + *Z*0_3_(*C*0^2^−*D*0^2^) = 0, the right side of Eq (11f) is purely imaginary (∴ *C0 =* 0):

$${Z0}_{1}-{Z0}_{3}{D0}^{2}=0$$ (31)

$$iD0=\frac{{Z0}_{0}}{i(2{\xi \omega \omega }_{po})}$$ (32)

Hence, the resonant frequency, *ω*_*po*_, can be obtained by solving Eqs (31–32).

Again, substitute Eq (17) into Eq (15) and collect the terms associated with *ɛ*^*1*^ to set up the following 1^st^ level equation:

$$\rho \frac{d^{2}{A1}_{Q}}{dt^{2}}+\rho \omega_{Q}^{2}{A1}_{Q}+3\beta_{Q}{{A0}_{Q}}^{2}{A1}_{Q}={R1}_{Q}(t)$$ (33)

where *R*1_*Q*_ is the sum of all terms associated with *ε*^*1*^.

Then, consider the harmonic balance of sin(*ωt*) to set up the following equation:

$$\int_{0}^{2\pi }{R1}_{Q}(t)\sin\left(\omega t\right)dt=0$$ (34)

$$-\rho \omega^{2}{A1}_{Q,1}+\rho \omega_{Q}^{2}{A1}_{Q,1}+\beta \left(\frac{9}{4}{{A0}_{Q,1}}^{2}{A1}_{1}-\frac{3}{4}{{{A0}_{Q,1}}^{2}A1}_{Q,3}\right)=0$$ (35)

Note that Eq (35) is a linear equation that contains two unknowns. *A*0_*Q*,*1*_ has been found at the zero level.

When substituting Eq (17) into Eq (15), collect those terms which contain the harmonic component of sin(3*ωt)*, to set up the following 1^st^ level equation (excluding those terms with *A*2_*Q*_ or higher level terms):

$$\rho \frac{d^{2}{A1}_{Q}}{dt^{2}}+\rho \omega_{Q}^{2}{A1}_{Q}+\beta_{Q}({{A0}_{Q}}^{3}+3{{A0}_{Q}}^{2}{A1}_{Q}+{3{A1}_{Q}}^{2}{A0}_{Q}+{{A1}_{Q}}^{3})={R1}_{Q,3}(t)$$ (36)

where *R*1_*Q*,*3*_ is the sum of the terms which contain the harmonic component of sin(3*ωt*).

Then, consider the harmonic balance of sin(3*ωt)* to set up the following equation:

$$\int_{0}^{2\pi }{R1}_{Q,3}(t)\sin\left(3\omega t\right)dt=0$$ (37)

$$-9\rho \omega^{2}{A1}_{Q,3}+\rho \omega_{Q}^{2}{A1}_{Q,3}+\beta_{Q}\left(\frac{-1}{4}{{A0}_{Q,1}}^{3}-\frac{3}{4}{{A0}_{Q,1}}^{2}{A1}_{Q,1}+\frac{3}{2}{{A0}_{Q,1}}^{2}{A1}_{Q,3}-\frac{3}{4}{{A1}_{Q,1}}^{2}{A0}_{Q,1}+3{A1}_{Q,1}{A1}_{Q,3}{A0}_{Q,1}-\frac{1}{4}{{A1}_{Q,1}}^{3}+\frac{3}{2}{{A1}_{Q,1}}^{2}{A1}_{Q,3}+\frac{3}{4}{{A1}_{Q,3}}^{3}\right)=0$$ (38)

By substituting Eq (35) into Eq (38), it can be expressed in the following cubic algebraic equation form, in terms of one unknown only:

$${Z1}_{0}+{Z1}_{1}{A1}_{Q,3}+{Z1}_{2}{{A1}_{Q,3}}^{2}+{Z1}_{3}{{A1}_{Q,3}}^{3}=0$$ (39)

where *Z*1_1_, *Z*1_2_, and *Z*1_3_ are the coefficients that precede the linear and nonlinear terms in Eq (38) and *Z*1_0_ is the zero-order term.

If damped cases are considered, rewrite eq (39) to obtain the following equation

$${A1}_{Q,3}=\frac{{Z1}_{0}}{{Z1}_{1}{+Z1}_{2}{A1}_{Q,3}+{Z1}_{3}{{A1}_{Q,3}}^{2}}$$ (40)

$$(C1+iD1)=\frac{{Z1}_{0}}{{Z1}_{1}+{Z1}_{2}C1+{Z1}_{3}({C1}^{2}-{D1}^{2})+i(6{\xi \omega \omega }_{p1}+{Z1}_{2}D1+2{Z1}_{3}C1D1)}$$ (41)

where *C1* and *D1* are the real and imaginary parts of *A1*_*Q*,*3*,_ respectively.

The 1^st^ level resonance occurs (i.e., the magnitude of *A1*_*Q*,*3*_ is maximum and *ω = ω*_p1_) in the frequency range at which the zero-level resonance does not occur. Thus, the damping term in *Z*1_0_ in Eq (41) can be omitted and the following expression is equal to 0

$${Z1}_{1}+{Z1}_{2}C1+{Z1}_{3}({C1}^{2}-{D1}^{2})=0$$ (42)

The right side of Eq (41) is purely imaginary (∴ *C1 =* 0):

$${Z1}_{1}-{Z1}_{3}{D1}^{2}=0$$ (43)

$$iD1=\frac{{Z1}_{0}}{i(6{\xi \omega \omega }_{p1}+{Z1}_{2}D1)}$$ (44)

Hence, the resonant frequency, *ω*_p1_ can be obtained by solving Eqs (43–44)

Again, substitute Eq (17) into Eq (15) and collect the terms associated with *ε*^*2*^ to set up the following 2nd-level equations:

$$\rho \frac{d^{2}{A2}_{Q}}{dt^{2}}+\rho \omega_{Q}^{2}{A2}_{Q}+3\beta_{Q}{{A1}_{Q}}^{2}{A0}_{Q}+3\beta_{Q}{{A0}_{Q}}^{2}{A2}_{Q}={R2}_{Q}(t)$$ (45)

where *R*2_*Q*_ is the sum of the terms associated with *ε*^*2*^.

Then, consider the harmonic balances of sin(*ωt*) and sin(3*ωt*)

$$\int_{0}^{2\pi }{R2}_{Q}(t)\sin\left(\omega t\right)dt=0$$ (46)

$$-\rho \omega^{2}{A2}_{Q,1}+\rho \omega_{Q}^{2}{A2}_{Q,1}+3\beta \left(\frac{3}{4}{A0}_{Q,1}{{A1}_{Q,1}}^{2}-\frac{1}{2}{A0}_{Q,1}{A1}_{Q,1}{A1}_{Q,3}+\frac{1}{2}{A0}_{Q,1}{{A1}_{Q,3}}^{2}+\frac{3}{4}{{A0}_{Q,1}}^{2}{A2}_{Q,1}-\frac{1}{4}{{A0}_{Q,1}}^{2}{A2}_{Q,3}\right)=0$$ (47)

$$\int_{0}^{2\pi }{R2}_{Q}(t)\sin\left(3\omega t\right)dt=0$$ (48)

$$-9\rho \omega^{2}{A2}_{Q,3}+\rho \omega_{Q}^{2}{A2}_{Q,3}+3\beta \left({{-\frac{1}{4}A0}_{Q,1}}^{2}{A2}_{Q,1}+\frac{1}{2}{{A0}_{Q,1}}^{2}{A2}_{Q,3}-\frac{1}{4}{{A0}_{Q,1}}^{2}{A2}_{Q,5}\right)=0$$ (49)

Note that the 1^st^ nonlinear term in Eq (45) has been used for the harmonic balance of sin(3*ωt*) in the 1^st^ level. Thus, it is not considered for the harmonic balance of sin(3*ωt*) in the 2^nd^ level. Eqs (47) and (49) are linear equations that contain three unknowns, as *A0*_*Q*_ and *A1*_*Q*_ have been found in the zero and 1^st^ levels.

When substituting Eq (17) into Eq (15), collect all terms with *A0*_*Q*_, *A1*_*Q*_ and *A2*_*Q*_, which contain the harmonic component of sin(5*ωt*), to set up the following 2nd-level equation:

$$\rho \frac{d^{2}{A2}_{Q}}{dt^{2}}+\rho \omega_{Q}^{2}{A2}_{Q}+\beta_{Q}(3{{A0}_{Q}}^{2}{A1}_{Q}+{3{A1}_{Q}}^{2}{A0}_{Q}+{{A1}_{Q}}^{3}+6{A0}_{Q}{A1}_{Q}{A2}_{Q}+{3{A2}_{Q}}^{2}{A0}_{Q}+{3{A1}_{Q}}^{2}{A2}_{Q}+{3{A0}_{Q}}^{2}{A2}_{Q}+{{A2}_{Q}}^{3})={R2}_{Q,5}(t)$$ (50)

where *R2*_*Q*,*S*_ is the sum of the terms with *A0*_*Q*,_, *A1*_*Q*,_ and *A2*_*Q*,_, which contain the harmonic component of sin(5*ωt*):

Then, consider the harmonic balance of sin(5*ωt*) to set up the following equation:

$$\int_{0}^{2\pi }{R2}_{Q,5}(t)\sin\left(5\omega t\right)dt=0$$ (51)

$$-25\rho \omega^{2}A2_{Q,5}+{\rho \omega }_{Q}^{2}A2_{Q,5}+\beta_{Q}(-\frac{3}{4}A0_{Q,1}{}^{2}A1_{Q,3}+\frac{3}{4}A0_{Q,1}A1_{Q,3}\left(A1_{Q,3}-2A1_{Q,3}\right)+\frac{3}{4}A1_{Q,1}A1_{Q,3}\left(A1_{Q,3}-A1_{Q,1}\right)+\frac{3}{2}A0_{Q,1}\left(A1_{Q,3}A2_{Q,3}-A1_{Q,1}A2_{Q,3}-A1_{Q,3}A2_{Q,1}+2A1_{Q,1}A2_{Q,5}\right)+\frac{3}{4}A0_{Q,1}\left(A2_{Q,3}{}^{2}-2A2_{Q,1}A2_{Q,3}+4A2_{Q,1}A2_{Q,5}\right)+3\left(\frac{-1}{4}A1_{Q,1}{}^{2}A2_{Q,3}+\frac{1}{2}A1_{Q,1}{}^{2}A2_{Q,5}-\frac{1}{2}A1_{Q,1}A1_{Q,3}A2_{Q,1}+\frac{1}{4}A1_{Q,3}{}^{2}A2_{Q,1}+\frac{1}{2}A1_{Q,1}A1_{Q,3}A2_{Q,3}+\frac{1}{2}A1_{Q,3}{}^{2}A2_{Q,5}\right)+\frac{3}{4}A0_{Q,1}{}^{2}\left(2A2_{Q,5}-A2_{Q,3}\right)+\left(\frac{3}{2}A2_{Q,1}{}^{2}A2_{Q,5}+\frac{3}{2}A2_{Q,1}A2_{Q,3}{}^{2}-\frac{3}{4}A2_{Q,1}{}^{2}A2_{Q,3}+\frac{3}{2}A2_{Q,3}{}^{2}A2_{Q,5}+\frac{3}{4}A2_{Q,5}{}^{3})\right)=0$$ (52)

By substituting Eqs (47) and (49) into Eq (52), it can be expressed as a cubic algebraic equation in terms of one unknown only

$${Z2}_{1}{A2}_{Q,5}+{Z2}_{2}{{A2}_{Q,5}}^{2}+{Z2}_{3}{{A2}_{Q,5}}^{3}={Z2}_{0}$$ (53)

where *Z2*_*1*_, *Z2*_*2*,_ and *Z2*_*3*_ are the coefficients that precede the linear and nonlinear terms. *Z2*_*0*_ is the constant term.

If damped cases are considered, rewrite Eq (15d) to obtain the following equation:

$${A2}_{Q,5}=\frac{{Z2}_{0}}{{Z2}_{1}{+Z2}_{2}{A2}_{Q,5}+{Z2}_{3}{{A2}_{Q,5}}^{2}}$$ (54)

$$(C2+iD2)=\frac{{Z2}_{0}}{{Z2}_{1}+{Z2}_{2}C2+{Z1}_{3}({C2}^{2}-{D2}^{2})+i(10{\xi \omega \omega }_{p2}+{Z2}_{2}D2+2{Z2}_{3}C2D2)}$$ (55)

where *C2* and *D2* are the real and imaginary parts of *A*2_Q,5_, respectively, and *ω*_*p*2_ is the resonant frequency of the 2nd-level resonance.

The 2nd-level resonance occurs (i.e., the magnitude of *A1*_Q,3_ is maximum and *ω* = *ω*_p1_) in the frequency range at which the zero level and 1st-level resonances do not occur. Thus, the damping term in *Z*2_0_ in Eq (55) can be omitted and the following expression is equal to 0.

$${Z2}_{1}+{Z2}_{2}C2+{Z1}_{3}({C2}^{2}-{D2}^{2})=0$$ (56)

Note that the right side of Eq (55) is therefore purely imaginary (∴ *C2 =* 0):

$${Z2}_{1}-{Z2}_{3}{D2}^{2}=0$$ (57)

$$iD2=\frac{{Z2}_{0}}{i(10{\xi \omega \omega }_{p2}+{Z2}_{2}D2)}$$ (58)

Hence, the resonant frequency *ω*_p2_ can be obtained by solving Eqs (57–58). Similarly, in the higher-level solution procedures, the harmonic components from the 3rd- or higher-level responses (e.g. *A3*_Q_ and *A4*_Q_) are used to set up the harmonic balance equations. One of these harmonic balance equations is a cubic algebraic equation and the others are linear. By solving these algebraic equations, the harmonic components of higher-level vibration amplitudes can be computed. For example, in the 3rd-level solution procedure, the three algebraic equations from the harmonic balances of sin(*ωt*), sin(3*ωt*), and sin(5*ωt*) are linear, whereas the one from the harmonic balance of sin(7*ωt*) is cubic. The unknowns in the four algebraic equations are *A3*_Q,1_, *A3*_Q,3_, *A3*_Q,5_, and *A3*_Q,7._ Using the three algebraic equations, *A3*_Q,1_, *A3*_Q,3_, and *A3*_Q,5_ can be expressed in terms of *A3*_Q,7._. Then substitute the expressions into the cubic algebraic equation to find *A3*_Q,7._ Once *A3*_Q,7_ is known, *A3*_Q,1,_ *A3*_Q,3_ and *A3*_Q,5_ can be determined using the three algebraic equations. Finally, the overall vibration amplitude of the *Q*^th^ mode is expressed as follows:

$$|A_{Q}|=\sqrt{\sum_{h=1,3,5\ldots }^{\bar{h}}{\left|A_{Q,h}\right|}^{2}}$$ (59)

where

$\bar{h}=highest\;solution\;level\;or\;harmonic\;order.$

After the nonlinear panel vibration amplitudes are obtained, the modal sound radiation and radiation efficiency can be computed using the modified Rayleigh’s integral method [29]:

$$\Lambda_{Q}=\int_{0}^{2\pi }\int_{0}^{\frac{\pi }{2}}\frac{{\left|\Xi_{Q}(r,\theta_{1},\theta_{2})\right|}^{2}}{\rho_{o}C_{a}}r^{2}sin(\theta_{1})d\theta_{1}d\theta_{2}$$ (60)

$$\sigma_{Q}=\frac{{8\Lambda }_{Q}}{\rho_{o}C_{a}a\prime b\prime }$$ (61)

$$\Xi_{Q}(r,\theta_{1},\theta_{2})=-ik_{h}\rho_{o}C_{a}\frac{e^{ik_{h}r}}{2\pi r}\int_{0}^{a\prime }\int_{0}^{b\prime }\phi_{Q}e^{-i(\frac{\eta_{x}x}{a\prime }+\frac{\eta_{y}y}{b\prime })}dxdy$$ (62)

where *r* is the distance between the panel corner and the observer point; *ϕQ* is the *Q*^th^ panel mode shape; θ_1_ and θ_2_ are the angles between the observer vector and y-axis and between the observer vector and *x*-axis, respectively (see [29] for details); *k*_*h*_ is the wave number; and *C*_*a*_ is the speed of sound.

Finally, the insertion loss is defined as the logarithm difference between the sound energy radiated from the nonlinear panel and the sound energy radiated from the source panel (i.e., the difference between the radiated sound levels in the cases with/without the nonlinear panel).

$$IL=-10\;log\;\left[\frac{\Lambda_{nlin}}{\Lambda_{sou}}\right]$$ (63)

$$\Lambda_{nlin}=\sum_{Q=1}^{\bar{Q}}\sigma_{Q}\rho_{o}C_{a}a\prime b\prime {\left|A_{Q}\right|}^{2}$$ (64)

$$\Lambda_{sou}=\sigma_{o}\rho_{o}C_{a}ab\;{\left|A_{o}\right|}^{2}$$ (65)

where

$\bar{Q}=number\;of\;panel\;modes\;used$
*σ*_*o*_ = radiation efficiency of the source panel mode.

## 3 Results and discussion

In this section, the material properties in the numerical cases considered are as follows: Young’s modulus = 7.1 × 10^10^ N/m^2^, Poisson’s ratio = 0.3 and mass density = 2,700 kg/m^3^. In Tables 4–6, the case is the nonlinear vibration of a single undamped panel subject to uniform excitation (i.e., no cavity).

**Table 4:** Vibration amplitude convergence for various excitation magnitudes (*ω* = *ω*_*1*_, *ξ* = 0.01, 2 structural modes, no cavity.

	*κ* = 0.1	= 0.5	= 1.2
Zero level Solution	100.55	101.88	104.35
1^st^ level Solution	100.02	100.16	100.79
2^nd^ level Solution	100.00*	100.00*	100.00*

*The 2^nd^ level values are normalized as 100).

**Table 5:** Vibration amplitude convergence for various excitation magnitudes(*ω* = 1.5*ω*_*1*_, *ξ* = 0.01, 2 structural modes, no cavity.

	*κ* = 0.1	= 0.5	= 1.2
Zero level Solution	101.35	101.80	102.55
1^st^ level Solution	100.06	100.11	100.23
2^nd^ level Solution	100.00*	100.00*	100.00*

*The 2^nd^ level values are normalized as 100).

**Table 6:** Vibration amplitude convergence for various excitation magnitudes(*ω* = 2*ω*_*1*_, *ξ* = 0.01, 2 structural modes, no cavity.

	*κ* = 0.1	= 0.5	= 1.2
Zero level Solution	101.75	101.94	102.26
1^st^ level Solution	100.09	100.12	100.17
2^nd^ level Solution	100.00*	100.00*	100.00*

*The 2^nd^ level values are normalized as 100).

The modal external excitation term in Eq (2), *F*_*Q*_, is expressed as *α*_Q_/*α*_QQ_ *κρg*, where *κ* is the dimensionless excitation parameter and *g* is 9.81 ms^-2^. The panel dimensions are 1 m × 1 m × 2 mm. The first two structural modes are used. Tables 4–6 show the convergence studies of normalised panel vibration amplitudes for various excitation magnitudes and frequencies. The 2nd-level solutions are normalised as 100. In the small excitation (*κ* = 0.1), the zero level solutions can achieve an error rate of less than 2% for the three different excitation frequencies. The 1st- and 2nd-level solutions to two–decimal place accuracy are almost identical. In the other two excitation cases (i.e., *κ* = 0.5 and *κ* = 1.2), the maximum difference between the zero-level and 2nd-level solutions is 4.35%, whereas the maximum difference between the 1^st^ and 2^nd^ level solutions is only 0.79%. It can be seen that the 1st-level solutions are good enough for three-digit accuracy. Tables 7 and 8 show the modal contributions of the nonlinear panel coupled with an extended cavity for various excitation magnitudes and frequencies. The panel dimensions are 1 m × 1 m × 3 mm. The cavity length and width are equal to 1.5 × the panel length. In each case, the sum of the three modal contributions is 100. Note that the 2nd and 4th modes are asymmetrical and are not considered for this symmetric load case. In the low-frequency excitations, the single-mode approach is sufficient because the contributions from the 3rd and 5th modes are minimal. When the excitation frequency is higher and closer to the resonant frequency of the 3rd mode (see the case of *ω* = 4 *ω*_3_ in Table 7), the 3rd-mode contribution is significantly higher and the 5th-mode contribution is still minimal. Because the frequency range considered in this study is 0 to 6 *ω*_1_, the two-mode approach is appropriate.

**Table 7:** Modal contributions for various excitation frequencies (*A*_o_/*τ* = 1, *ξ* = 0.01, cavity depth = panel length).

	*ω/ ω*_*1*_ = *1*	= 2	= 4
1^st^ mode	99.98	100.00	78.52
3^rd^ mode	0.02	0.00	21.43
5^th^ mode	0.00	0.00	0.05

**Table 8:** Modal contributions for various source panel amplitudes (*ω* = *ω*_*1*_, *ξ* = 0.01, cavity depth = panel length).

	*A*_o_/*τ* = 0.5	= 1	= 2
1^st^ mode	99.99	99.98	99.96
3^rd^ mode	0.01	0.02	0.04
5^th^ mode	0.00	0.00	0.00

**Fig 3:**
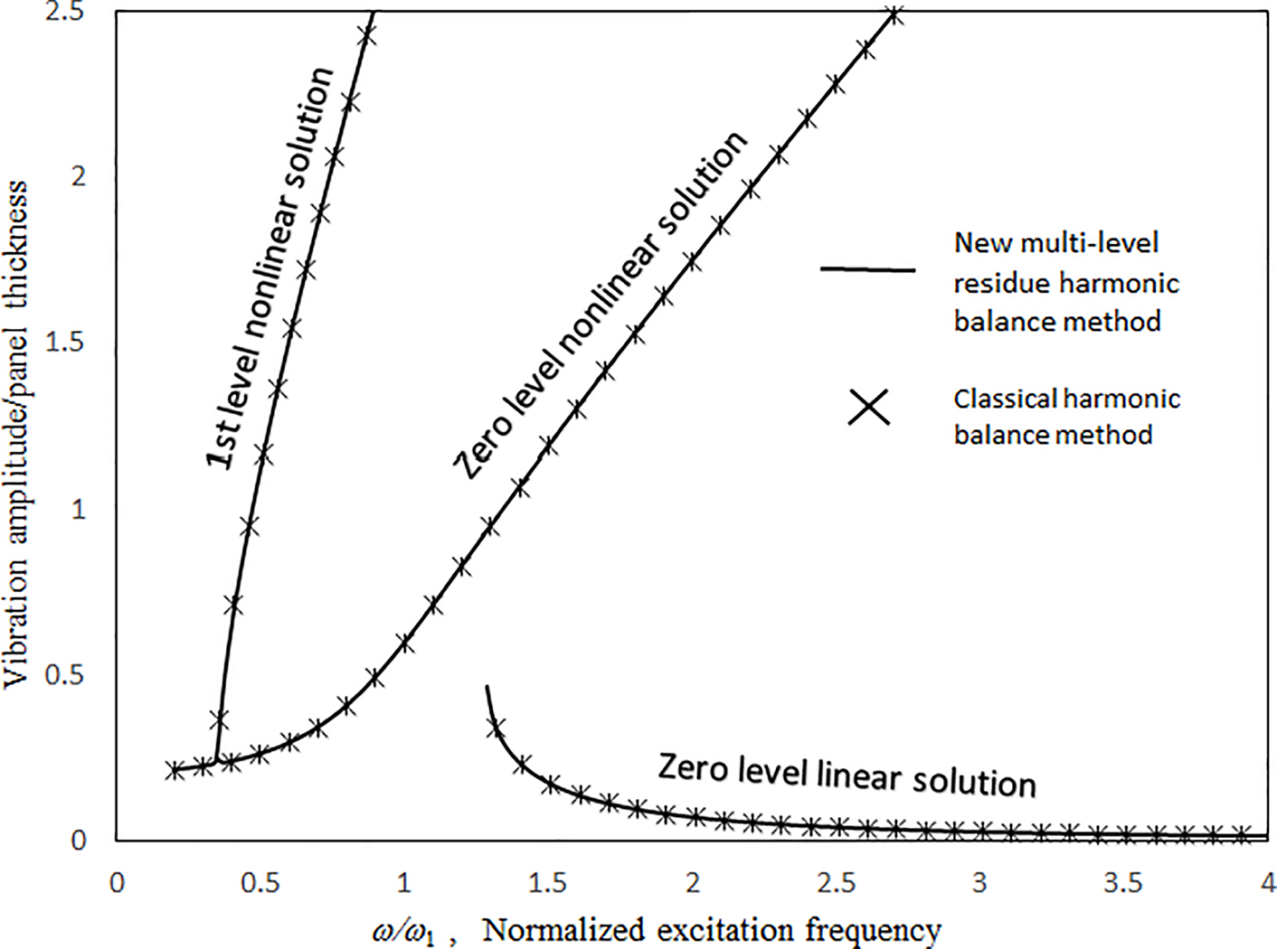
Comparison of the undamped vibration amplitude results from the proposed and classical harmonic balance methods (*τ* = 2 mm, *a = b* = 1m, *κ* = 0.1, no cavity).

**Fig 4:**
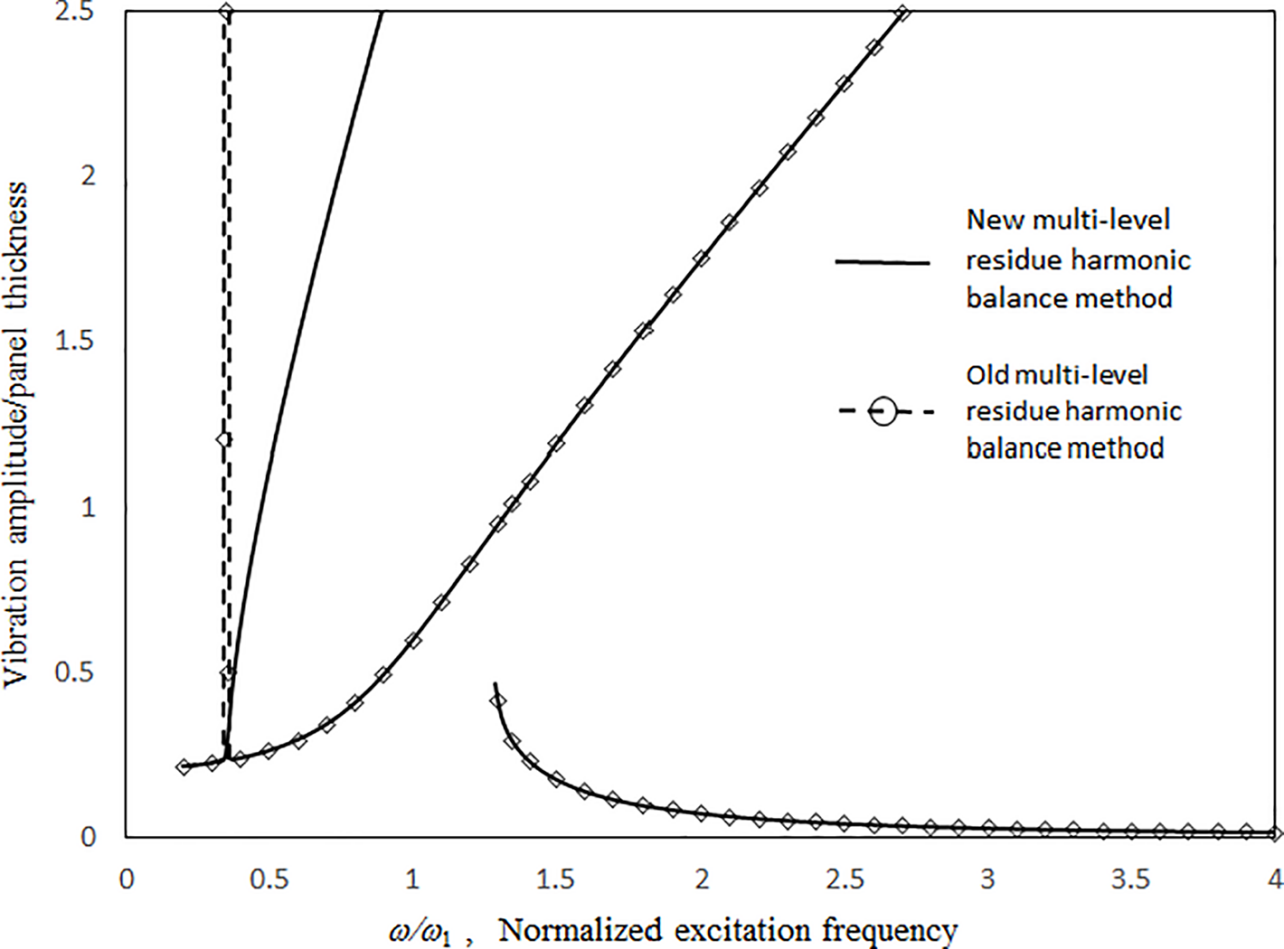
Comparison of the undamped vibration amplitude results from the proposed and old multi-level residue harmonic balance methods (*τ* = 2 mm, *a = b* = 1m, *κ* = 0.1, no cavity).

**Fig 5:**
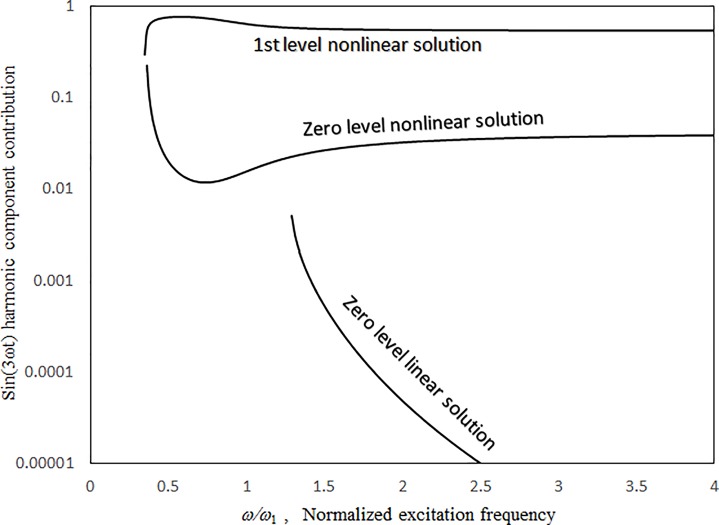
Sin(3*ω*t) harmonic component contribution in Fig 2A versus normalized excitation frequency (*τ* = 2 mm, *a = b* = 1m, *κ* = 0.1, no cavity).

**Fig 6:**
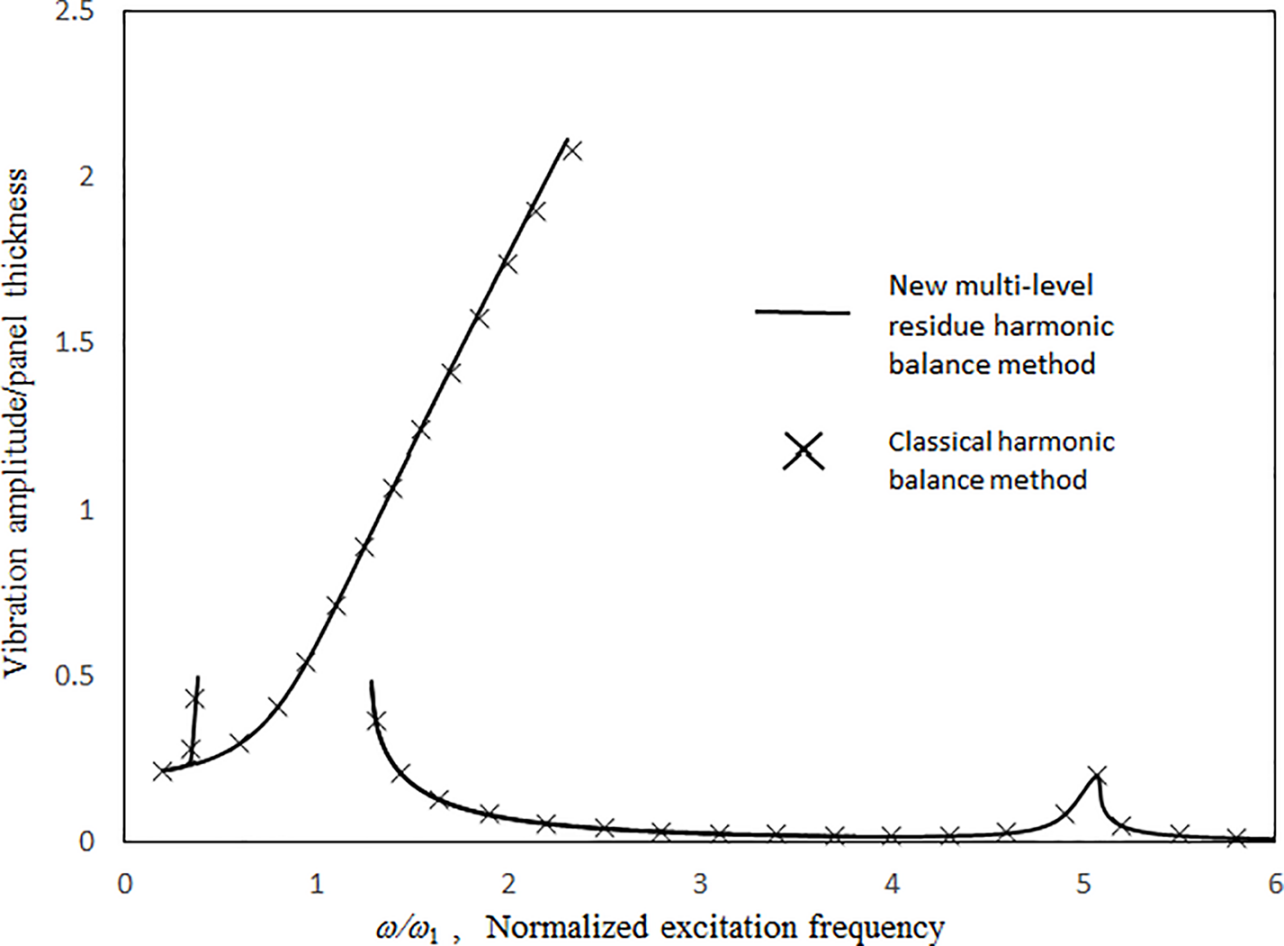
Comparison of the damped vibration amplitude results from the proposed and classical harmonic balance methods (*τ* = 2 mm, *a = b* = 1m, *κ* = 0.1, ξ = 0.01, no cavity).

Figs 3 and 4 present the comparisons between the 1st-mode vibration amplitudes of a single undamped panel subject to uniform excitation (i.e., no cavity) obtained with the proposed classical harmonic balance method [31] and the previous multilevel residue harmonic balance method [32,33]. The dimensionless excitation parameter value *κ* = 0.1. In Fig 3, the results obtained from the two methods are generally in good agreement for both zero- and 1st-level nonlinear solutions. In Fig 4, the 1st-level solution of the previous multilevel residue harmonic balance method is linear and much different from those from the proposed method and the classical harmonic balance method. Only linear equations are set up in the 1st level of the previous multilevel residue harmonic balance method. Fig 5 shows the contribution of sin(3*ωt*) component to the vibration response in Fig 3. First, in the simple harmonic solution line, the contribution of sin(3*ωt*) component is very minimal. It is more like a linear solution. Second, in the zero-level nonlinear solution line, the contribution of sin(3*ωt*) component is detectable, but not dominant. Third, in the 1st level nonlinear solution line, the contribution of sin(3*ωt*) component is very significant. It is more like a super-harmonic solution. Fig 6 shows the comparisons between the two-mode vibration amplitudes of a single damped panel subject to uniform excitation, which are obtained from the proposed method and classical harmonic balance method. The dimensionless excitation parameter value *κ* = 0.1. The results obtained from the two methods are generally in good agreement, except for the resonant peak values around *ω = 0*.*6 ω*_*Q*_ and *ω = 2*.*2 ω*_*Q*_. The peak value differences are due to the complete coupling of the damping terms amongst all nonlinear equations in the classical harmonic balance method, whilst the damping terms are uncoupled from one solution level to higher solution levels in the proposed method. The 3rd-mode resonant peak values from the two methods are also in good agreement because the resonant peaks are more linear.

**Fig 7:**
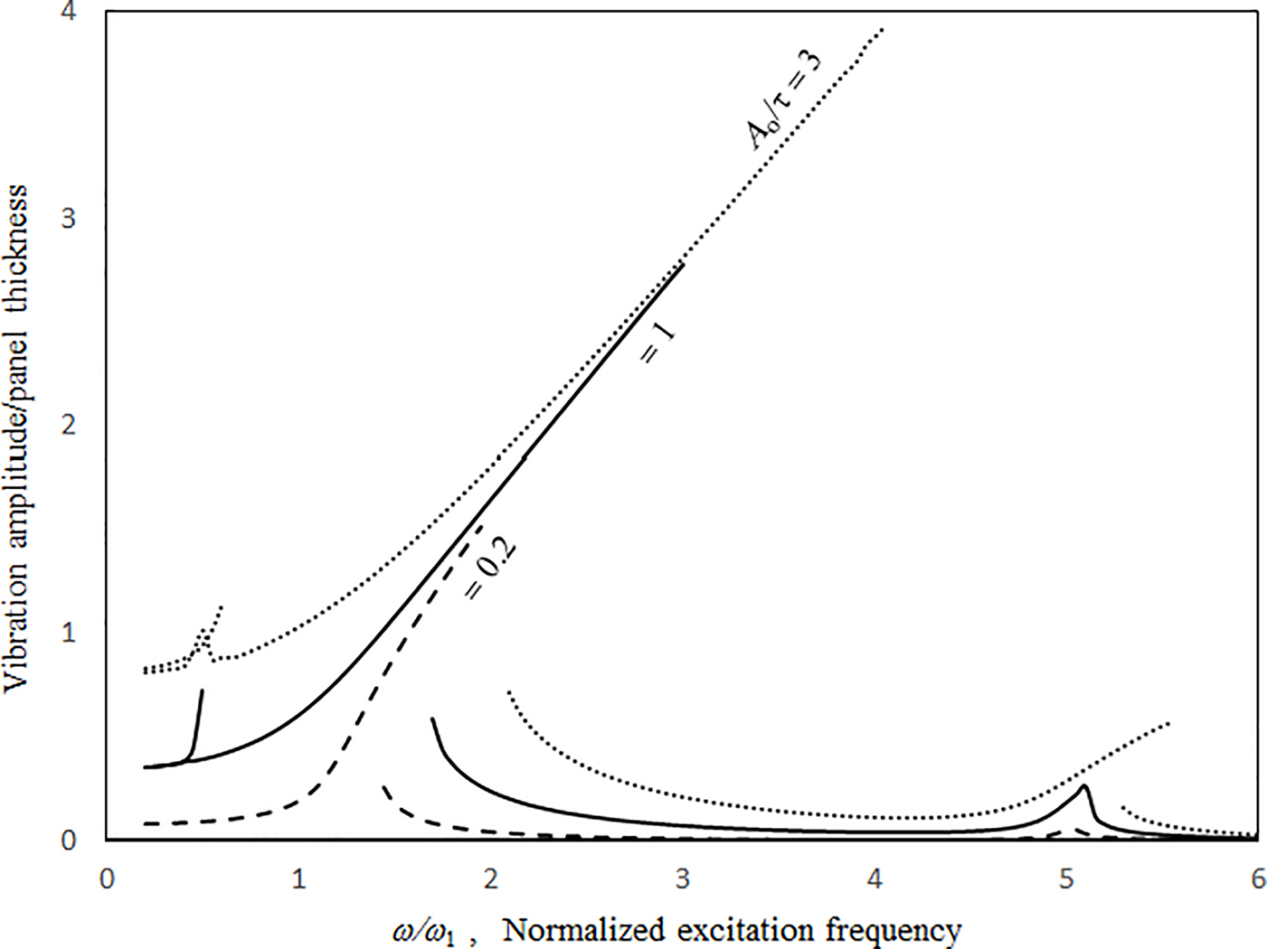
Vibration amplitude versus excitation frequency for various source excitation magnitudes (*τ* = 3 mm, *a’ = b’* = *c* = 1m, *a = b =* 1.5 *a’*, *ξ* = 0.01).

**Fig 8:**
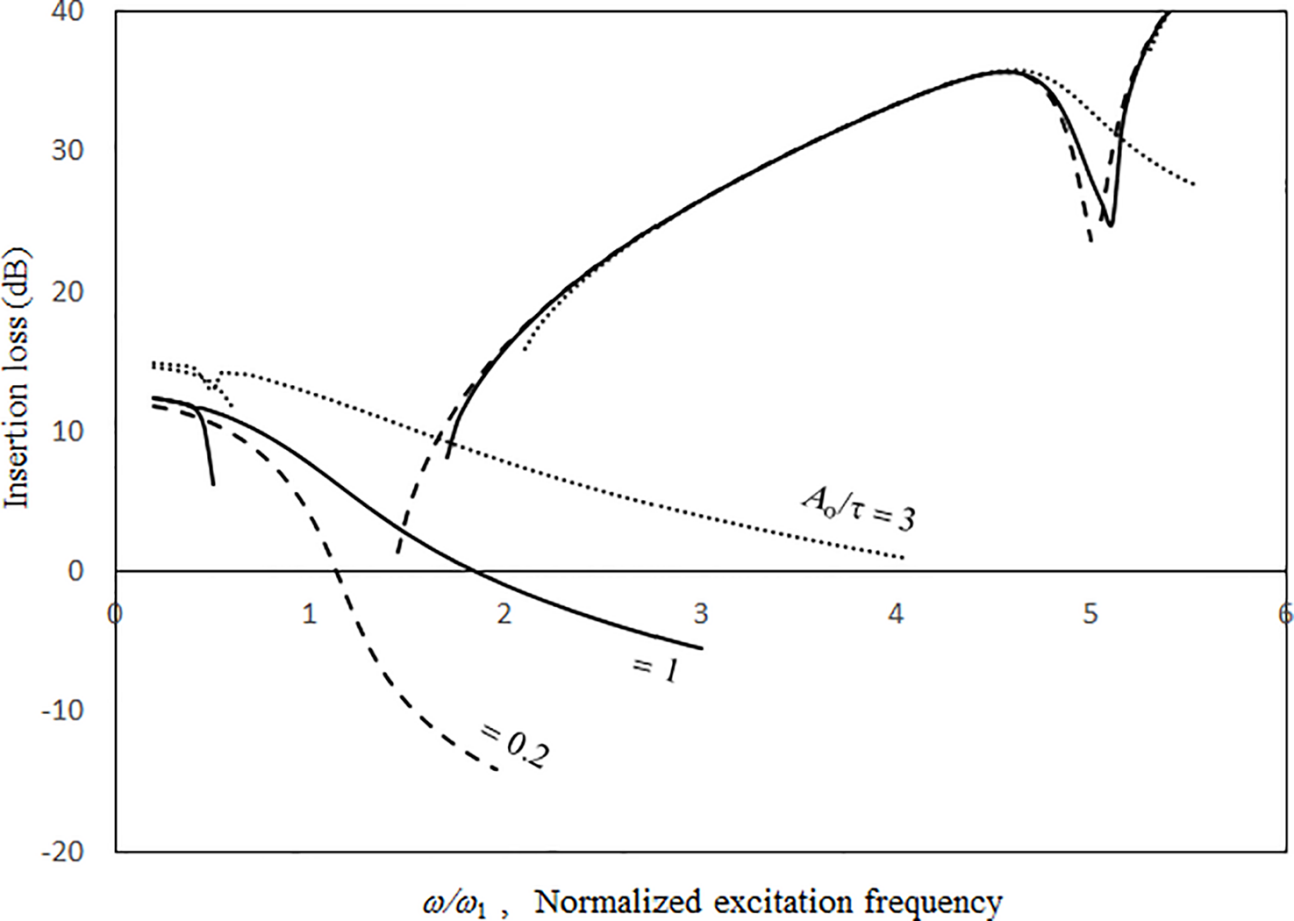
Insertion loss versus excitation frequency for various source excitation magnitudes (*τ* = 3 mm, *a’ = b’* = *c* = 1m, *a = b =* 1.5*a’*, *ξ* = 0.01).

Figs 7 and 8 show the vibration amplitude and insertion loss plotted against the excitation frequency for various source excitation magnitudes. In Fig 7, the peak frequencies and vibration amplitude increase with the excitation magnitude. The peaks are caused by the structural resonances. It is noted that when it is very small excitation, the normalised resonant frequency of the panel without cavity effect is one. The peak frequencies in Fig 7 are higher than one because of the structural nonlinearity and cavity stiffness. The acoustic resonant frequencies are outside the frequency range concerned in Figs 7 and 8. The jump phenomenon occurs at approximately *ω/ω*_*l*_ = 3. The higher the excitation level, the higher the degree of nonlinearity and the higher the peak frequency that can be seen at the vibration peak. The three solution lines converge closely around the zero-level nonlinear resonance. The slope of the solution line of 3*τ* around the zero-level nonlinear resonance is the shallowest, whilst the slope of the solution line of 0.2*τ* varies sharply (i.e., from shallow to deep). Generally, the 1st-mode vibration peaks are much higher than the 3rd-mode vibration peaks. The 3rd-mode vibration peak of 0.2*τ* is very small and more symmetric than that of high excitation. Furthermore, there is no jump phenomenon at the 3rd-mode vibration peak and no 1st-level nonlinear resonance. Unlike that in Fig 7, the higher the excitation level or degree of nonlinearity, the higher the insertion loss dip value that can be seen in Fig 8. In the non-resonant frequency ranges from *ω/ω*_*l*_ = 2.5 to *ω/ω*_*l*_ = 4.5, the three curves in Fig 8 are very close. Figs 7 and 8 show that the resonant bandwidths are wider for higher excitation magnitudes (note that it is a negative effect on the insertion loss of an acoustic panel). Thus, the linear deign of an acoustic panel may incorrectly estimate the noise reduction performance under high excitation.

**Fig 9:**
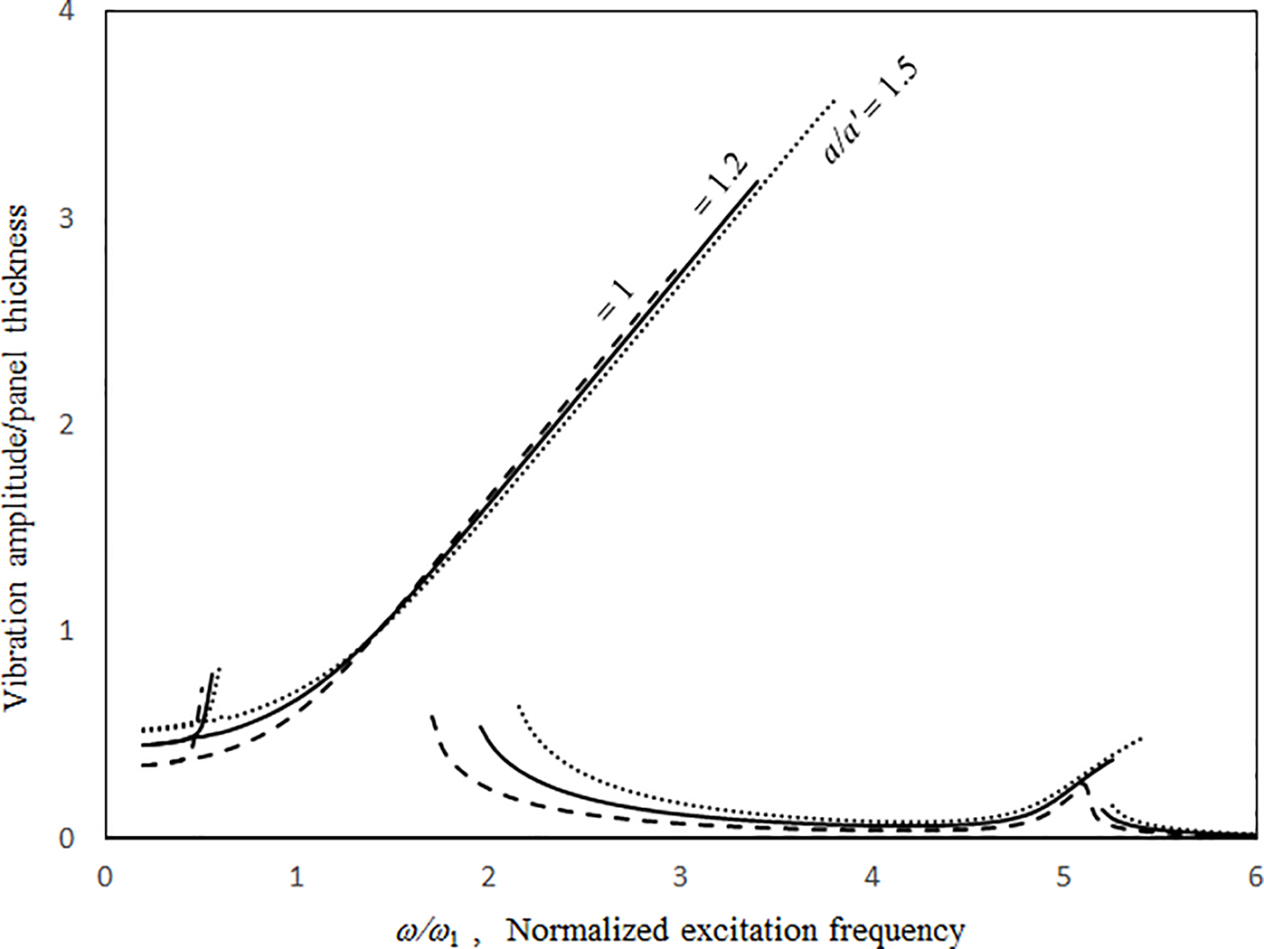
Vibration amplitude versus excitation frequency for various cavity lengths (*τ* = 3 mm, *a = b*, *a’ = b’ = c* = 1m, *A*_o_/*τ* = 1, *ξ* = 0.01).

**Fig 10:**
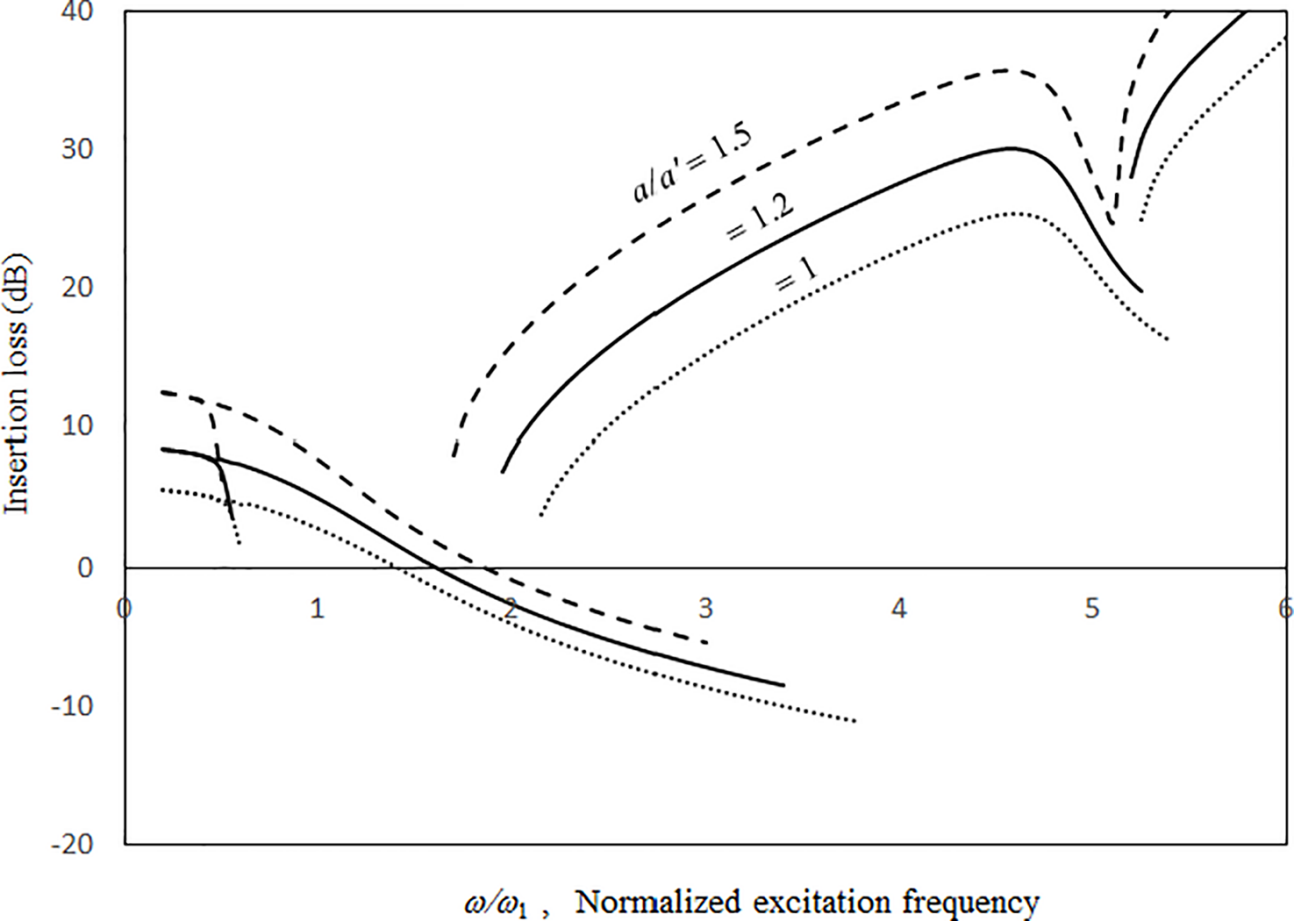
Insertion loss versus excitation frequency for various cavity lengths (*τ* = 3 mm, *a = b*, *a’ = b’ = c =* 1m, *A*_o_/*τ* = 1, ξ = 0.01).

**Fig 11:**
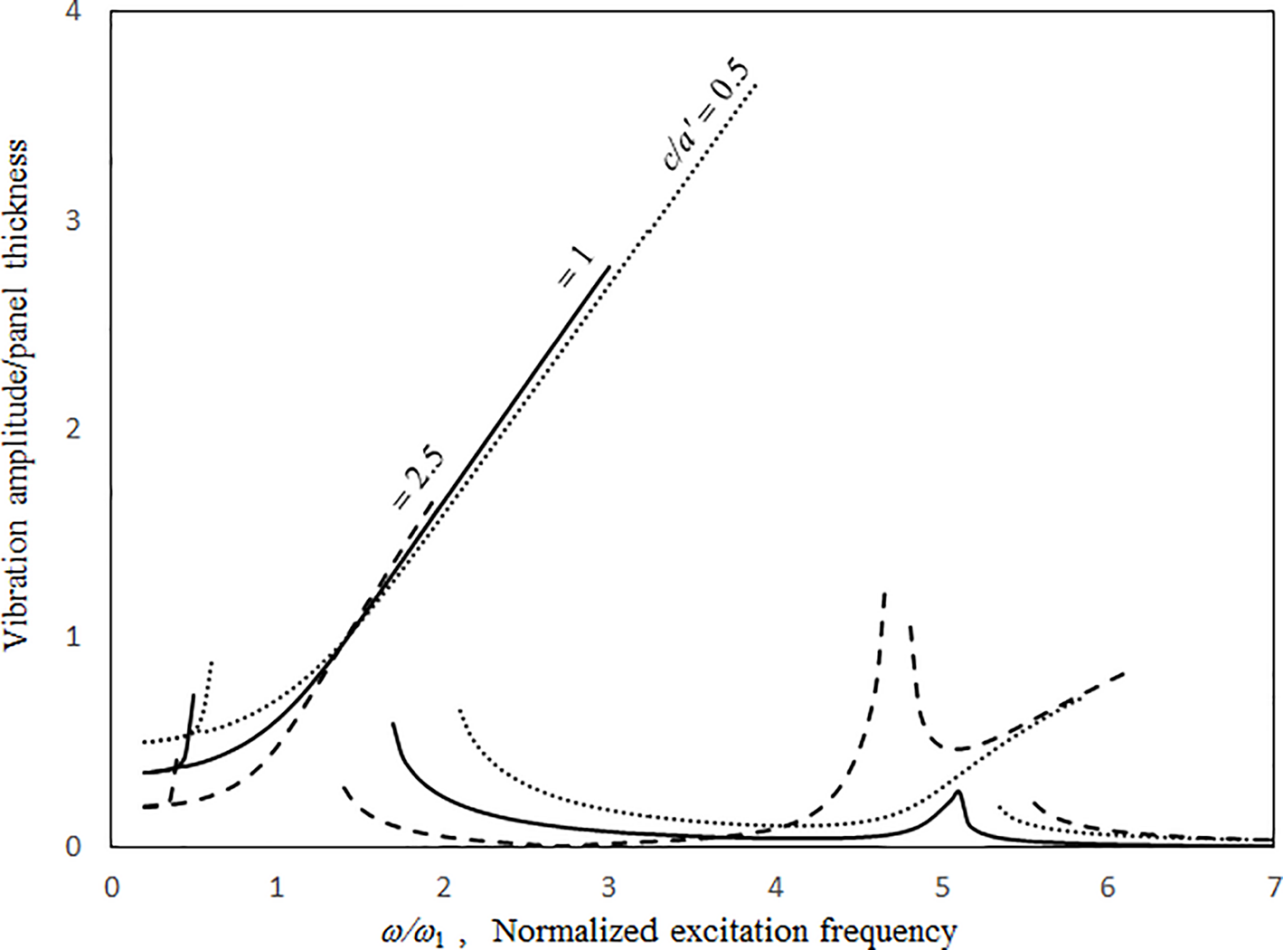
Vibration amplitude versus excitation frequency for various cavity depths (*τ* = 3 mm, *a’ = b’ =* 1.5m, *a = b* = 1.5*a’*, *A*_o_/*τ* = 1, ξ = 0.01).

**Fig 12:**
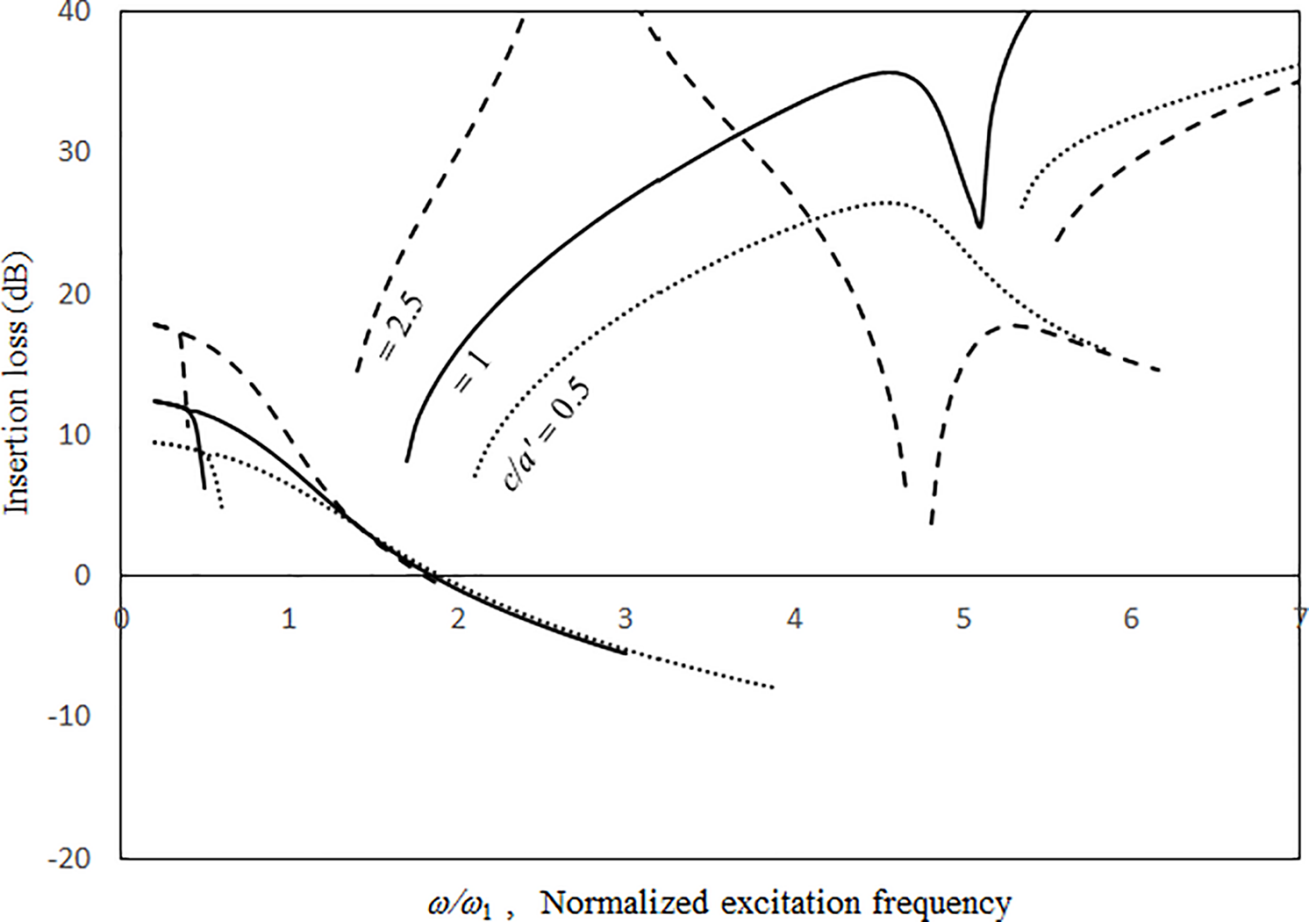
Insertion loss versus excitation frequency for various cavity depths (*τ* = 3 mm, *a’ = b’ =* 1.5m, *a = b* = 1.5*a’*, *A*_o_/*τ* = 1, ξ = 0.01).

**Fig 13:**
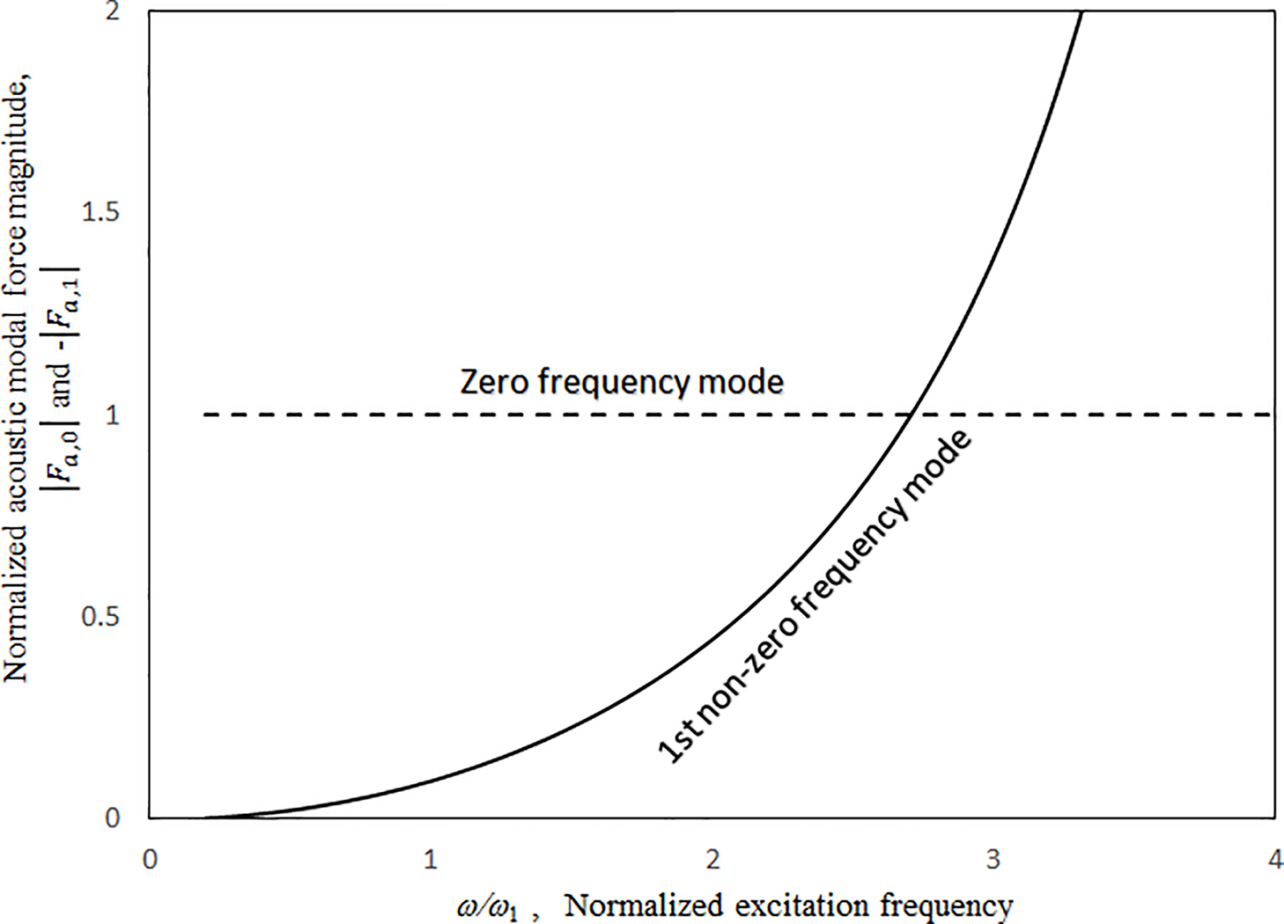
Acoustic modal force magnitude versus excitation frequency(*τ* = 3 mm, *a’ = b’ =* 1.5m, *a = b* = 1.5*a’*, *A*_o_/*τ* = 1, ξ = 0.01).

Figs 9–12 show the vibration amplitudes and insertion losses plotted against the excitation frequency for various cavity lengths and depths. The zero- and 1st-level nonlinear peak amplitudes/insertion loss dip values and frequencies decrease with the cavity length and depth. Unlike those in Fig 7 around the zero-level resonance, the three curves in Figs 9 and 11 are very close but intercept at approximately *ω/ω*_*l*_ = 1.5. When *ω/ω*_*l*_ < 1.5, the curves of *a = a’* and *c* = 0.5*a’* are the highest in Figs 9 and 11, respectively. When *ω/ω*_*l*_ > 1.5, the curves of *a = a’* and *c* = 0.5*a’* are the lowest. The 1st-level nonlinear resonance is not significantly affected by changing the cavity dimensions. It is obvious that the nonlinearity of the structural acoustic system depends greatly upon the cavity size. If the cavity size is smaller, the nonlinearity is higher. A large cavity volume implies a low stiffness or small acoustic pressure transmitted from the source panel to the nonlinear panel. Unlike those in Fig 8, in the non-resonant frequency ranges from *ω/ω*_*1*_ = 2.5 to *ω/ω*_*l*_ = 4.5, the three curves in Figs 10 and 12 are clearly separate. The 2nd structural resonance occurs from *ω/ω*_*l*_ = 5.0 to *ω/ω*_*1*_ = 5.5 in the three cases in Figs 9–10 and the case of *c* = *a’* in Figs 11 and 12, whereas the 2nd structural resonance occurs around *ω/ω*_*l*_ = 5.8 to *ω/ω*_*1*_ = 6.2 for the cases of *c* = 0.5*a’* and *c* = 2.5*a’* in Figs 11 and 12. As mentioned, in the case of *c* = 0.5*a’* in Figs 11 and 12, the smaller cavity results in a higher nonlinearity so that the resonant peak/insertion loss dip and the corresponding peak and dip frequencies are higher. In the case of *c* = 2.5*a’* in Figs 11 and 12 (note that the cavity depth is very long and it looks like a tube), although the cavity is much bigger, the nonlinear phenomenon (i.e., jump phenomenon) around the 2nd structural resonance is obvious. The 1st acoustic resonance around *ω/ω*_*1*_ = 4.7, which is close to and strongly coupled with the 2nd structural resonance, amplifies the nonlinearity. Note that in the case of *c* = 2.5*a’* in Fig 5A and 5B, there is no solution found from *ω/ω*_*l*_ = 4.7 to 4.8 and thus the solution line is discontinuous there. In Fig 12, there is an anti-resonant peak at approximately *ω/ω*_*l*_ = 2.7 in the case of *c* = 2.5*a’*. The acoustic pressure forces of the zero-frequency cavity mode and the 1st non-zero frequency cavity mode (their mode numbers are *l*_*x*_ *= l*_*y*_ *= l*_*z*_ = 0 and *l*_*x*_ *= l*_*y*_ *=* 0; *l*_*z*_ = 1) acting on the nonlinear panel are opposite. Fig 13 shows the two normalised acoustic modal force magnitudes (i.e., |*F*_*a*,0_| and -|*F*_*a*,1_|) against the normalised excitation frequency. The two curves intercept at approximately *ω/ω*_*l*_ = 2.7.

**Fig 14:**
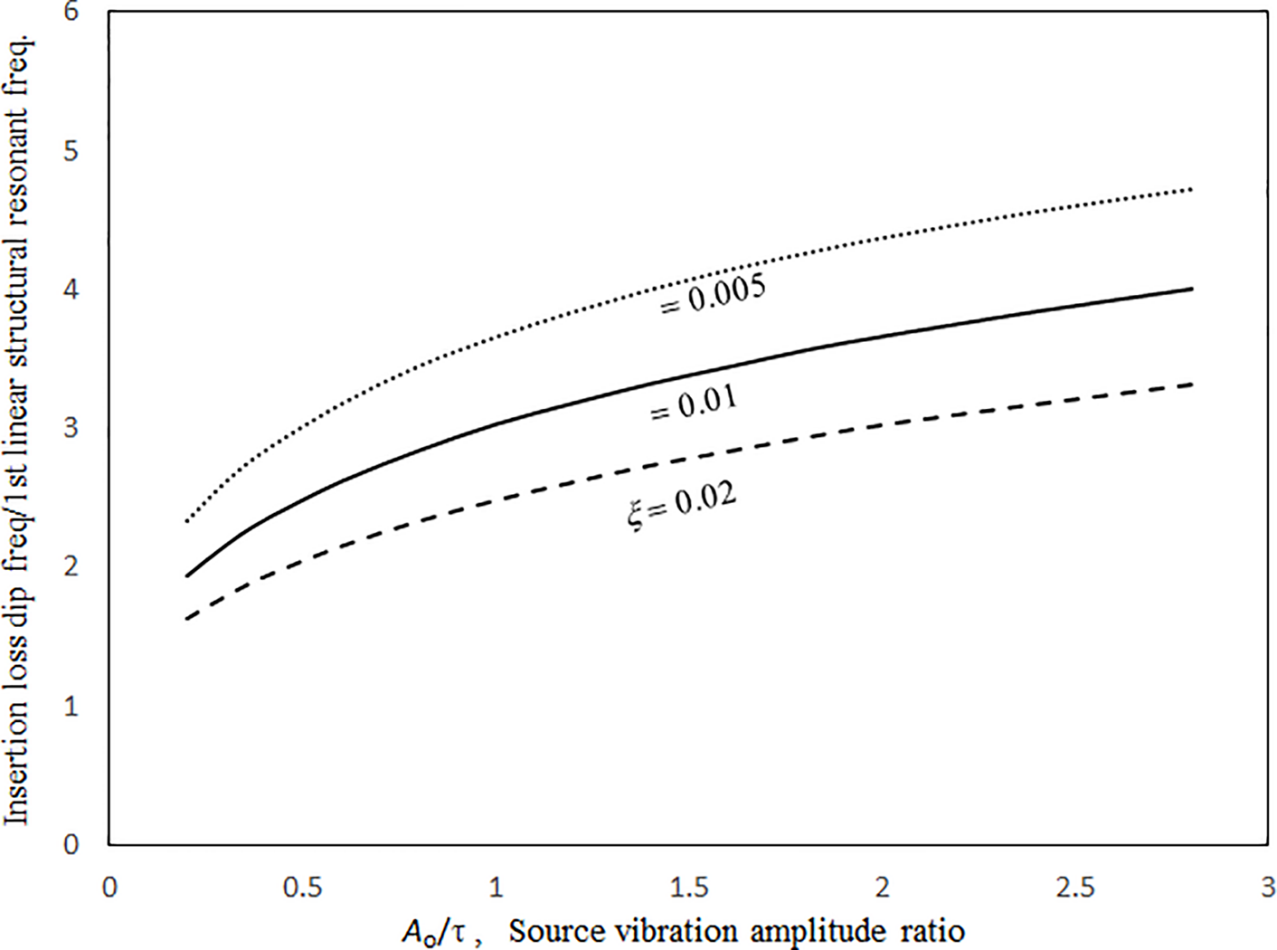
Insertion loss dip frequency versus source vibration amplitude ratio for various damping ratios (*τ* = 3 mm, *a’ = b’ = c* = 1m, *a = b =* 1.5*a’*).

**Fig 15:**
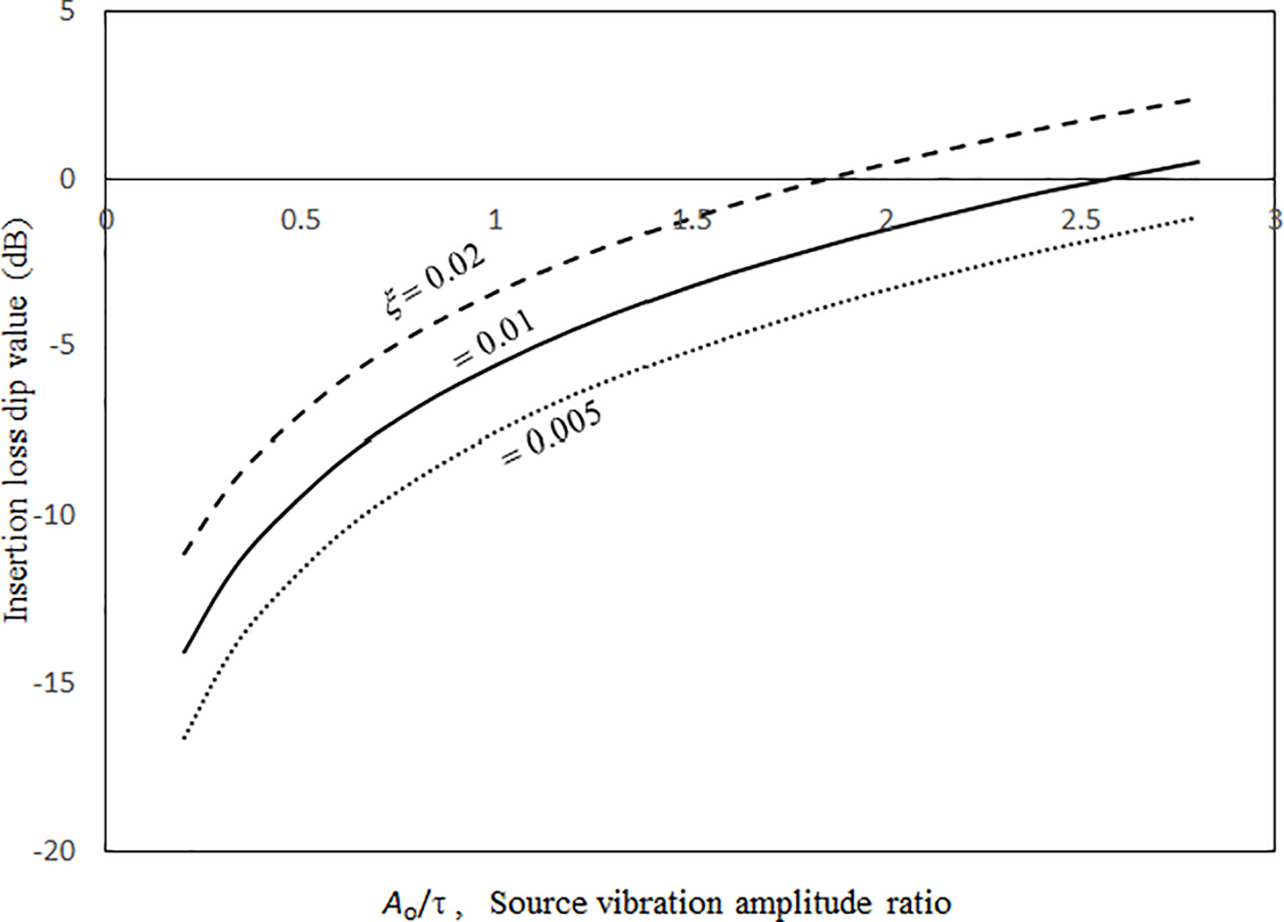
Insertion loss dip value versus source vibration amplitude ratio for various damping ratios (*τ* = 3 mm, *a’ = b’ = c =* 1m, *a = b =* 1.5*a’*).

**Fig 16:**
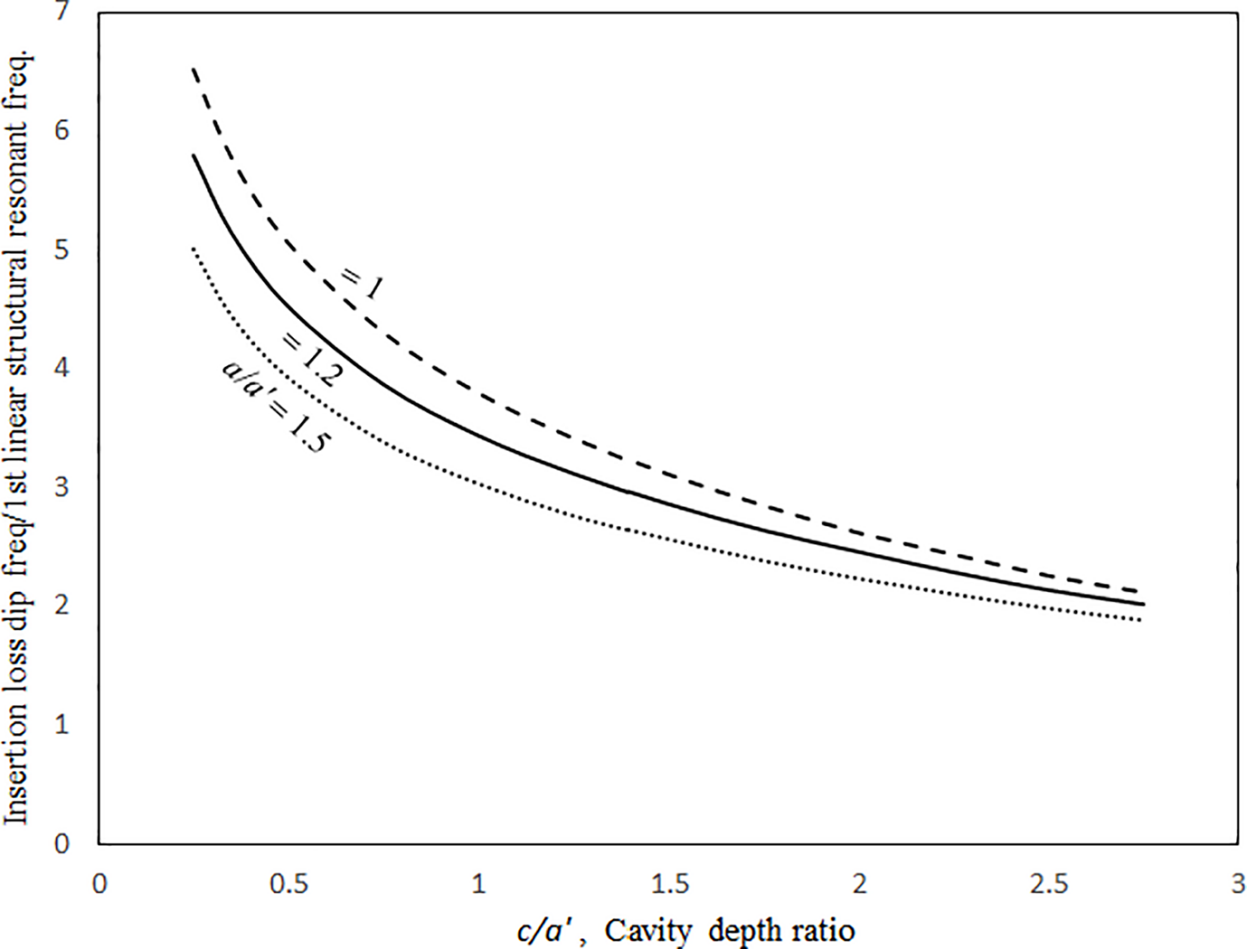
Insertion loss dip frequency versus cavity depth ratio for various cavity lengths (*τ* = 3 mm, *a’ = b’ =* 1m, *a = b*, *A*_o_/*τ* = 1, ξ = 0.01).

**Fig 17:**
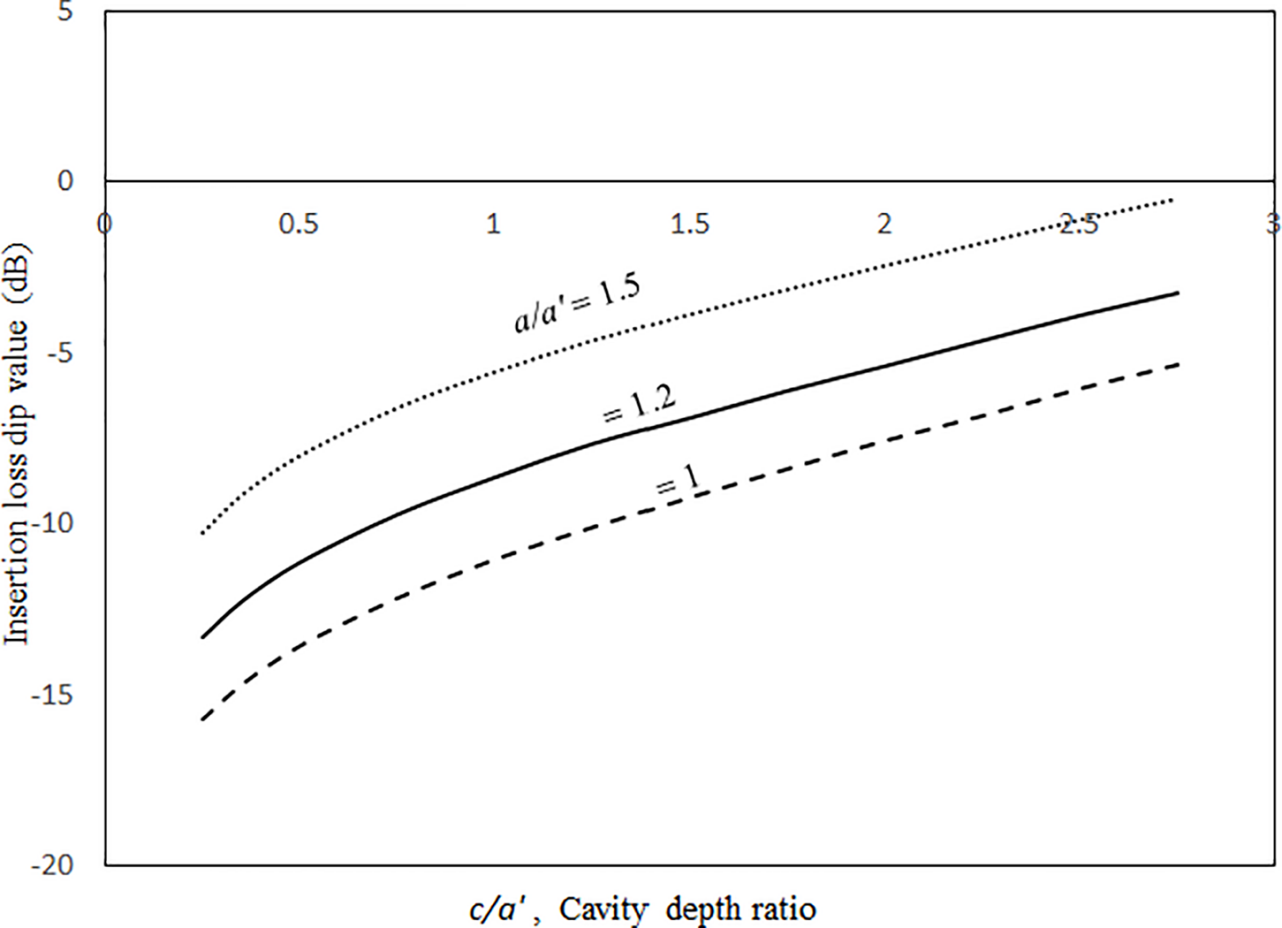
Insertion loss dip value versus cavity depth ratio for various cavity lengths (*τ* = 3 mm, *a’ = b’ =* 1m, *a = b*, *A*_o_/*τ* = 1, ξ = 0.01).

Figs 14 and 15 show the insertion loss dip frequencies and values plotted against the excitation magnitude for various damping ratios. It can be seen that the insertion loss dip values and the corresponding dip frequencies of the three cases increase monotonically with the excitation magnitude. When the excitation magnitude is low, the insertion loss dip frequencies in the three cases converge and the slopes of the three curves deepen. A low excitation magnitude results in linear panel vibrations in the system; the resonant frequency is not significantly affected by the damping. When the excitation magnitude is high, the three curves are almost linear and far from each other. Unlike the resonant frequency, the insertion loss dip value always highly depends on the damping ratio in the system. Thus, the three dip value curves are separate for the entire range of excitation magnitude. Figs 16 and 17 show the insertion loss dip frequencies and values plotted against the cavity depth for various cavity lengths. The insertion loss dip frequencies and values decrease and increase with the cavity depth, respectively. It is found that if the cavity volume is bigger (i.e., the depth or length is greater), the insertion loss is higher. Furthermore, if the cavity depth is longer, the dip frequencies of the three cases get close. Finally, if the dip frequencies are lower, then the corresponding insertion loss values are higher. A larger cavity results in a smaller nonlinearity or a lower resonant frequency in the system. Thus, the vibration amplitude or the insertion loss dip value is higher.

## 4 Conclusions

This study analyses the insertion loss of a nonlinearly vibrating panel backed by an extended cavity. The proposed harmonic balance method is applied to this nonlinear structural acoustic problem. The structural/acoustic modal formulation has been developed from partial differential equations, which represent the large amplitude structural vibration of a flexible panel coupled with an extended cavity. The results obtained from the proposed harmonic balance method and classical harmonic balance method are generally consistent. The effects of excitation magnitude, damping ratio, cavity depth and length are investigated. The results show that the nonlinearity of a structural acoustic system depends greatly upon the cavity size. If the cavity size is smaller, the nonlinearity is higher. A large cavity volume implies a low stiffness or small acoustic pressure transmitted from the source panel to the nonlinear panel. Thus, the additional volume in an extended cavity would affect the nonlinearity, sound and vibration responses of a structural acoustic system. Furthermore, if acoustic resonance couples with structural resonance, the nonlinearity is amplified, adversely affecting the insertion loss.

## Supporting information

S1 File Data PLOS. (XLSX)
